# Laser Powder-Bed Fusion of Ceramic Particulate Reinforced Aluminum Alloys: A Review

**DOI:** 10.3390/ma15072467

**Published:** 2022-03-27

**Authors:** Tatevik Minasyan, Irina Hussainova

**Affiliations:** Department of Mechanical and Industrial Engineering, Tallinn University of Technology, Ehitajate 5, 19086 Tallinn, Estonia

**Keywords:** laser powder-bed fusion, additive manufacturing, aluminum alloys, reinforcement, ceramic particulates, grain refinement, crystallographic texture, mechanical properties

## Abstract

Aluminum (Al) and its alloys are the second most used materials spanning industrial applications in automotive, aircraft and aerospace industries. To comply with the industrial demand for high-performance aluminum alloys with superb mechanical properties, one promising approach is reinforcement with ceramic particulates. Laser powder-bed fusion (LPBF) of Al alloy powders provides vast freedom in design and allows fabrication of aluminum matrix composites with significant grain refinement and textureless microstructure. This review paper evaluates the trends in in situ and ex situ reinforcement of aluminum alloys by ceramic particulates, while analyzing their effect on the material properties and process parameters. The current research efforts are mainly directed toward additives for grain refinement to improve the mechanical performance of the printed parts. Reinforcing additives has been demonstrated as a promising perspective for the industrialization of Al-based composites produced via laser powder-bed fusion technique. In this review, attention is mainly paid to borides (TiB_2_, LaB_6_, CaB_6_), carbides (TiC, SiC), nitrides (TiN, Si_3_N_4_, BN, AlN), hybrid additives and their effect on the densification, grain refinement and mechanical behavior of the LPBF-produced composites.

## 1. Introduction

In many engineering solutions, product performance is determined by weight, which can be scaled down by material-efficient construction and the use of low-density alloys [1,2]. Due to exceptional strength/stiffness-to-weight ratio, low density, good damage tolerance, ability to be heat treated and the low cost, aluminum (Al) alloys are extensively used in many exclusive fields, such as: automotive, aerospace, marine navigation, rail transit, architectural construction, microelectronics and consumer applications [3,4,5,6,7].

In the meantime, owing to the moderate strength and relatively poor wear resistance of aluminum alloys, they are not applicable as structural materials for critical parts of aircrafts or satellites [8,9]; therefore, there is a need to improve the mechanical properties of aluminum alloys to be used for special applications. Along the modern industrial developments, the demand for complex-shaped products in diverse sectors is widespread. Problems related to traditional casting of aluminum alloys include coarse microstructures, a long process chain with limited flexibility [10], use of PM/casting molds [11] and a high rate of tool degradation [12].

Additive manufacturing (AM) provides an integrated way of item production [13]. Additive manufacturing, also known as 3D printing, refers to the layer-wise fabrication process of functional objects adopting nearly unlimited geometrical complexity, processing freedom, high level of accuracy and customization with elimination of traditional economy-of-scale constraints [14]. Furthermore, the material efficiency and design flexibility of AM technology meet the requirements for resource optimization, mass customization and accelerates the time to enter the market. In terms of dissimilar material joining and hybrid structures, AM is considered a versatile tool for complete spatial control of local material composition, microstructure and properties [15].

Among the most advanced AM technologies available, laser powder-bed fusion has gained increased attention in both the industrial and academic sectors. The essence of the process lies beneath the selective melting/solidification of the desired sections of consecutive powder layers by a precise (computer-controlled) high-energy laser beam directed by 3D CAD (computer-aided design) file [16,17,18]. Within the scanning process, the laser energy is supplied into the powder layer, and the powder particles–laser beam interaction takes place over a very short duration resulting in high heating/cooling rates [19,20,21]. The heat is absorbed by the powder particles following both bulk coupling and powder coupling mechanisms [11]. The laser-aided processing not only produces layers of fused powder, but also creates metallurgical bond with its preceding layer, which leads to a proper densification and competent mechanical behavior of the fabricated parts. Generally, the LPBF process can be ascribed with the following steps: scattering and absorption of laser waves by the powder particles, heat transfer, melting and coalescence of particles, generation of the melt pool and its solidification [22,23]. Due to a high cooling rate (up to 10^6^ K/s), the microstructure of the fabricated samples can dramatically differ from the conventionally prepared counterparts [3,24]. During solidification, the melted material tends to undergo a significant non-equilibrium metallurgical process, demonstrating different modes of heat and mass transfer, causing the formation of unique microstructures [25].

During the laser treatment, each powder layer possesses its innate thermal history, generating a complex thermal cycle, which results in high residual stresses, periodic cracks, undesirable microstructural features and a lack of morphological uniformity [26]. Intricate physics governing the laser beam–feedstock interaction (energy absorption, heat and mass transfer), in situ chemical reactions, phase transformations and lack of insights of uncontrollable non-equilibrium metallurgical processes restrict the printability of many alloys by LPBF [13,27]. To date, most commercial aluminum alloys for important applications remain challenging for processing by LPBF due to feedstock particles’ poor flowability, high affinity to oxygen, high laser reflectivity (hence low absorptivity), high material thermal conductivity, large solidification range and solidification cracking [4,10,14]. The 2xxx, 6xxx and 7xxx series of high-strength age-hardenable aluminum alloys contain elements that widen the solidification temperature range, leading to the segregation of phases with low melting point during epitaxial grain growth [28]. Moreover, the high thermal conductivity and high laser reflectivity of materials require excess heat to reach melting. This can cause vaporization of volatile alloying elements (Zn, Mg, etc.) and lead to heterogeneity within the completed part [10]. Hence, alloys with a large solidification range have a poor applicability to AM due to the formation of hot cracks at various process stages [23].

There are several near-eutectic Al–Si alloy grades suitable for LPBF and available on the market. These materials display an excellent fluidity, high thermal conductivity, low coefficient of thermal expansion (CTE) and outstanding castability [29]. Hypoeutectic Al–Si (7–12 wt.%)-Mg (>1 wt.%) alloys [10,30] possess the largest share among Al alloys applicable for LPBF process. The incorporation of silicon is a critical issue for Al–Si alloys, since Si reduces the melting point and narrows the solidification temperature range through the formation of a eutectic, thus inhibiting crack formation and propagation. Nevertheless, LPBF-fabricated Al–Si alloys generally face issues of low strength, low ductility, moderate fatigue and wear resistance, which limit their use as structural components [4,8], and, hence, there is an admitted necessity to develop novel aluminum alloys for LPBF. Owing to extremely quick solidification process inherent to LPBF, the majority of high-strength alloys, traditionally esteemed to be “non-weldable materials”, suffer from hot cracking and porosity along the columnar grain boundary. However, even so determined “printable” alloys through LPBF possess a non-uniform microstructure and demonstrate poor mechanical performance [31].

**Figure 1:**
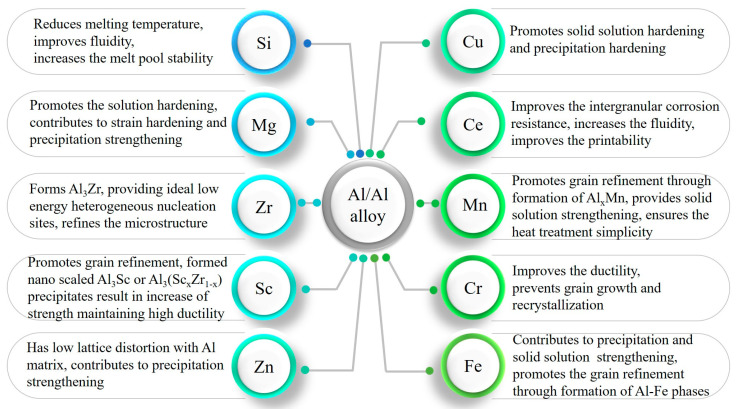
The influence of the main and modifying components on LPBF fabricated Al alloys [14,27,30,33,38,39,40,41,42,43,44,45,46,47,48,49,50,51,52,53,54].

For a wide acceptance of the alloys for industrial use, the materials must ensure a number of required properties. The ideal alloy must be highly matched for the extreme thermal conditions by means of decreasing fabrication defects. Meanwhile, it is crucial for it to possess a suitable microstructure along with specific mechanical properties, which are comparable to the existing peak-aged wrought alloys, and to maintain a major part of its strength at elevated or high temperatures [30]. To further improve the mechanical performance of LPBF-prepared aluminum alloys, a substantial amount of research has been devoted to the following:

(i) Studying the modification of existing compositions by minor alloying constituents to generate strengthening phases upon the fabrication process or during post-processing (heat treatment) [32]. (The effects of common modifying elements are given in Figure 1).
(ii) The addition of grain refiners (stable, non-soluble solid ceramic particulates) to reduce hot-tear susceptibility, grain growth and dislocation motion by developing aluminum matrix composites (AMC) [8,33]. The latter conveys a combination of properties of two or more physically distinct phases with the aim to produce parts with far superior properties to the individual components [34].
(iii) Heat treatment [35,36,37].

AM processes are categorized as master forming technologies, where customized designed objects’ properties are generated by the fabrication process itself. Therefore, the composition and aluminum alloy chemistry undertake a central role during the LPBF process [1]. Combining the advantages offered by AM with the favorable mechanical properties of aluminum alloys will create viable mass-market manufacturing strategies that will increase the adoption and implementation of both across the world [7].

In this review paper, the focus is placed on the laser powder-bed fusion of the ceramic particulate (boride, carbide, nitride and hybrid additive) reinforced aluminum alloys, concentrating on the effect of additives on the microstructure and grain refinement of the produced materials. Thereafter, the mechanical properties and the mechanisms responsible for their change are confronted to lead to a deeper understanding of the possible performance of ceramic particulate reinforced aluminum matrix composites (AMCs). The list of used reinforcements and their unique features during the LPBF process, as well as diagrams showing the strengthening, hardening and grain-refining effect of the added particulates, are specified. The properties and efficiency of AMCs prepared by the traditional or other additive manufacturing techniques are beyond the scope of this paper.

### Reinforcement with Ceramic Particulates

The influence of rapid cooling during LPBF on the Al alloy microstructure is described by three factors: (i) constitutional changes due to a great level of undercooling; (ii) individual phase refinement, when the scale of microstructural refinement is strongly related to the velocity of the solidification interface; (iii) generation of phases in metastable state [10].

In contrast to coarse-grained cast Al alloys, LPBF-fabricated Al alloys exhibit a refined microstructure, reduced dendritic branching, decreased segregation patterns, extensions of solid solubility of alloying components, formation of metastable crystalline, quasi-crystalline, amorphous phases [10] and microstructural anisotropy [55].

Generally, the anisotropy in LPBF-fabricated parts is a major processing bottleneck triggered by the generation of coarse columnar grains with a preferential crystallographic texturing along the build direction [56]. The main microstructural characteristics in LPBF-fabricated hypoeutectic Al–Si alloys are columnar primary-Al grains and the eutectic Si phase. The formation of such columnar grains is induced by the high thermal gradients, which hinders nucleation ahead of the solidification front stimulating epitaxial grain growth during LPBF [57]. Epitaxially grown columnar grains are formed during partial (or complete) melting of the preceding solidified layers upon laser scanning of new layers and further develop through successive irradiated layers. Moreover, the formation of columnar grains can lead to intergranular hot tearing [58]. An effective solution is to provoke the equiaxed grain formation during cooling process, which is reached upon modulating the thermal gradient, cooling rate and alteration of cooling conditions [59,60].

One of the approaches for microstructure and properties optimization during LPBF processing is either ex situ or in situ inoculation. In situ reactions in the particle-reinforced composite systems prohibit the formation of interfacial compounds, support the nucleation and growth from the parent matrix phase to generate chemically more stable reinforcing compounds. The distribution of the in situ reinforcements is more homogeneous and provides a strong interfacial bonding with the matrix [61]. The chemical reaction between the reactants might also originate an extra thermal energy for the fusion, which can strengthen the matrix-reinforcement binding. Such assets lead to supreme material performances, allowing MMCs (metal matrix composites) to reach mechanical properties far superior to the ex situ reinforced or non-reinforced metals/alloys. However, due to a wide variety of technological challenges, these MMCs are seldom implemented for commercial applications. Successful design requires a large number of factors to be considered, such as powder compositions, presence of native oxide films on powder particles, powder flow, exothermicity of the in situ reaction and process parameters. The “in situ” formed elements, such as O, C and N, might dissolve in a metal matrix, causing significant embrittlement. Furthermore, additional heat released during the process might cause melt pool instability, leading to an intensive powder splash and evaporation [62,63].

Commonly, for grain refinement, the addition of stable grain refiners (inoculants) with the smallest possible lattice mismatch to aluminum is widely used in conventional casting processes. Refiners suppress the columnar solidification and promote the formation of a fine, uniform, equiaxed grain structure by stimulating heterogeneous nucleation and achieving the columnar-to-equiaxed transition [64]. The latter magnifies the total area of grain boundaries per unit volume, decreasing the residual liquid film thickness along the solidification process, and thus prohibits the formation and propagation of cracks [28]. The heterogeneous nucleation of α-Al during solidification takes place preferably on the inoculants, which provide the low-energy interfaces between a refiner and a matrix [65].

To determine the comparative values of interfacial energy, atomic matching throughout the interface is generally employed as an indicator. To reduce interfacial energy, the main requirements are coherent or semi-coherent interfaces and reproducible orientation relationships (ORs) between two crystals, as different lattice parameters cause distortion of the lattice, resulting in an excess strain energy, which is determined by a lattice mismatch (also called as lattice disregistry, *δ*) [58]. The selection of potent grain refiners with the smallest disregistry with the matrix crystal throughout a specific interface is favored [58]. If disregistry value is below 10%, both in situ formed and added inoculants have the ability to induce heterogeneous nucleation of Al grains [66].

Nucleant particles serve a dual role in the AMCs as refiners and reinforcements, and they can be classified in three categories: non-oxide ceramics, oxide ceramics and carbon-based compounds. Generally, the ceramic particulates of a high hardness, good thermal stability, relatively high laser absorptivity and compatibility with metals/alloys are suitable constituents for the preparation of high-performance AMCs [67]. To meet the demand to satisfy the “light weight and high strength” concept, novel AMCs are continuously under development [5,11,68].

For the conventional AMCs, relatively coarse ceramic particles with a size ranging from several tens to hundreds of micrometers are broadly utilized as reinforcements. However, reasoned by limited interfacial wettability between reinforcement and matrix, the large particles are susceptible to cracking during mechanical loading, causing reduced ductility and inducing premature failure of AMCs [69]. Consequently, both tensile strength and ductility of AMCs increase if the fine-sized reinforcements are used. On that account, the introduction of the nano-scaled ceramic particles can remarkably enhance the mechanical performance of AMCs [70,71].

However, the agglomeration of nanoparticles may cause unfavorable microstructural changes and affect the mechanical behavior of the composites, as well as affecting thermal and rheological behavior of the melt pool (increasing viscosity, especially in case of high volume of nanoparticles) and shifting the LPBF parameter window. The LPBF method enables effective fabrication of composites reinforced with ceramic reinforcements, taking into account the unique metallurgical nature of the process, high temperatures and thermal convection in a micron-sized molten pool [23,72,73].

## 2. Non-Oxide Additives

**Figure 2:**
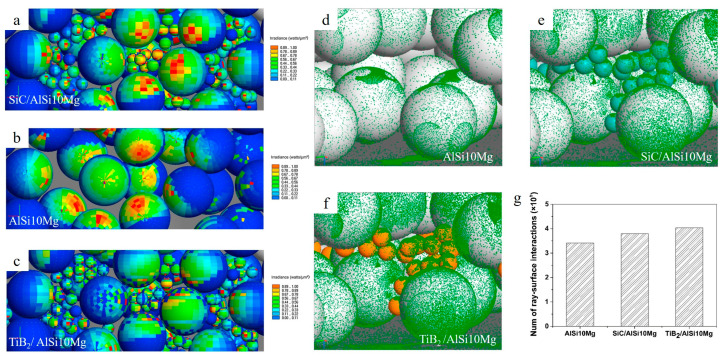
Irradiance distribution for SiC/AlSi10Mg (**a**), AlSi10Mg (**b**), TiB_2_/AlSi10Mg (**c**) powder mixtures (top view). Illustration of track spot of each laser ray on the particle surface of AlSi10Mg (**d**), SiC/AlSi10Mg (**e**), TiB_2_/AlSi10Mg (**f**) (side view) and numerical representation of laser–particle interactions (**g**) (reproduced with permission from [76]).

Non-oxide additives (borides, carbides, nitrides, etc.) are one of the most used reinforcements for Al alloys due to their high melting temperatures and chemical stability [74]. AMCs merge the ductility and toughness of aluminum with the high strength and modulus of the ceramic reinforcement [75], hence achieving an improvement of the overall characteristics and durability [12]. The low laser absorptivity of aluminum in the infrared range challenges the controlled melting, while the increase in the laser absorption of ceramic particulate decorated/mixed aluminum alloy at a laser wavelength of 1064 nm promotes the LPBF process. The introduction of ceramic particles to the pure alloy increases laser absorptivity of the overall powder mixture, as (i) non-oxide ceramic particles display high laser absorptivity and (ii) the added ceramic particles increase surface roughness of decorated powder, promoting multiple reflections of the laser in the powder bed [28]. As shown in Figure 2a–c, the ray absorption of the SiC/AlSi10Mg and TiB_2_/AlSi10Mg powder mixtures is higher compared to pure AlSi10Mg alloy. There is a lower intensity of interactions between laser rays and particles of pure AlSi10Mg compared to SiC and TiC added composite powder. (Figure 2d–g) [76].

### 2.1. Borides: Grain Refining and Strengthening Effect of TiB_2_, LaB_6_, CaB_6_

As one of the proven highly effective grain refiners for Al alloy, TiB_2_ particles exhibits good thermal stability, good wettability and interfacial compatibility, in addition to the acknowledged crystallographic orientation relationship with Al matrix, contributing to a comprehensive mechanical performance of AMCs [59,73]. The addition of TiB_2_ to AlSi10Mg increases the laser absorptivity of the powder bed by almost 1.5 times [76]. To provide even distribution, small particle size and adequate interfacial bonding of the TiB_2_ particles, in situ fabrication approaches have been implemented, offering the advantages of a clean interface between ceramic particles and matrix alloy and fine morphology of in situ formed particles [5]. Both in situ and ex situ fabrication of TiB_2_ reinforced Al alloys are discussed below.

**Figure 3:**
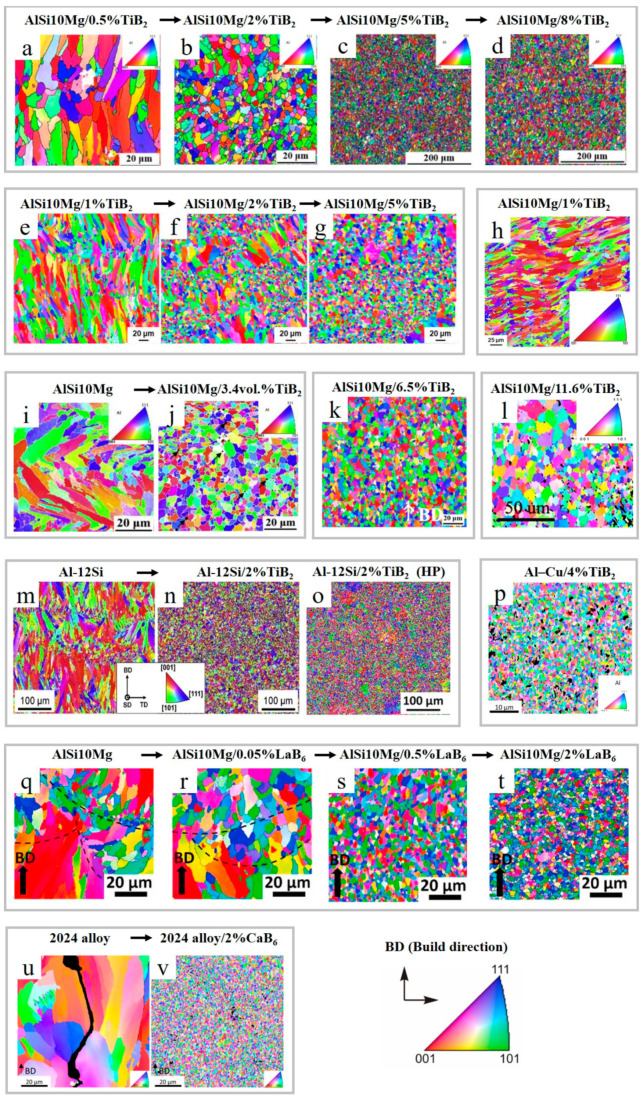
EBSD (electron backscatter diffraction) color maps for LPBF-prepared Al alloys and AMCs reinforced with borides ((**a**–**n**) and (**p**–**v**)) (subfigure (**o**) represents hot-pressed (HP) sample) (reproduced with permission from [28,59,64,73,77,78,79,80,83,84]).

In Ref. [77], 0.5–8 wt.% nano-sized TiB_2_ particles were introduced into AlSi10Mg, which resulted in the elimination of columnar grains and refined elongated dendritic structures from 4.6 to 2 µm, as shown in Figure 3a–d and Table 1. Similar results were obtained in Refs. [59,73], as the introduction of 1–5 wt.% and 5.3 wt.% (3.4 vol%) TiB_2_ to AlSi10Mg, respectively, led to remarkable grain refinement down to 1.55 µm (Figure 3e–g,i,j). However, the incorporation of only 1 wt.% TiB_2_ into AlSi10Mg [78] did not demonstrate a dramatic difference between reinforced and pure alloy parts; however, the grain size distribution became distinctly narrow (Figure 3h).

**Table 1:** Characteristics of boride (particulate) reinforced AMCs fabricated by laser powder-bed fusion.

System	Used Device, Process Parameters	Relative Density (%)	Average Grain Size (μm)	σ_y_/σ_u_ (MPa)	ε/ε_c_ (%)	Hardness (HV)	N
AlSi10Mg/ 1 wt.% TiB_2_	SLM 150 HL P = 350–450 W ν = 1800 mm/s d = 50 μm h = 50 μm E_v_ = 77.7–100.0 J/mm^3^	99.95	~6.3	-	-	~126 HV0.2	[78]
AlSi10Mg/ 3.4 vol.%TiB_2_	Prox DMP 200 SLM P = 210 W ν = 1000 mm/s d = 30 μm h = 100 μm E_v_ = 70 J/mm^3^	99.975	2.08	σ_u_ = 522.9–529	ε ≈ 7.5–8.6	-	[59]
AlSi10Mg/ 1 wt.%TiB_2_	SLM 150 P = 450 W ν = 1600–2600 mm/s d = 50 μm h = 50 μm E_v_ = 69.2–112.5 J/mm^3^	Up to 99.09	6.32 ± 0.07	σ_y_ ≈ 270 σ_u_ = 397	ε ≈ 3.6	~124 HV0.2	[73]
AlSi10Mg/ 2 wt.% TiB_2_		Up to 99	2.20 ± 0.11	σ_y_ ≈ 283 σ_u_ ≈ 444	ε ≈ 4.2	~127 HV0.2	
AlSi10Mg/ 5 wt.% TiB_2_		~96–97.8	1.55 ± 0.14	σ_y_ ≈ 270 σ_u_ = 422	ε ≈ 4.1	~129 HV0.2	
AlSi10Mg	Prox DMP 200, 3D Systems P = 220–280 W ν = 800–2000 mm/s d = 30 μm h = 90 μm	99.56 ± 0.16	4.64	σ_y_ = 270.1 ± 4.3 σ_u_ = 430.7 ± 1.6	ε = 4.7 ± 0.4	125.9 ± 1.4 HV10	[77]
AlSi10Mg/ 0.5 wt.% TiB_2_		99.82 ± 0.10	3.45	σ_y_ = 317.6 ± 2.1 σ_u_ = 484.1 ± 3.3	ε = 9.5 ± 0.3	140.5 ± 1.3 HV10	
AlSi10Mg/ 2 wt.% TiB_2_		99.92 ± 0.04	2.0	σ_y_ = 320.1 ± 3.2 σ_u_ = 500.7 ± 3.5	ε = 12.7 ± 0.2	147.1 ± 1.5 HV10	
AlSi10Mg/ 5 wt.% TiB_2_		99.91 ± 0.02	~2.0	σ_y_ = 323.7 ± 1.9 σ_u_ = 522.9 ± 3.6	ε = 8.7 ± 0.5	151.1 ± 2.1 HV10	
AlSi10Mg/ 8 wt.% TiB_2_		99.92 ± 0.05	~2.0	σ_y_ = 340.8 ± 1.7 σ_u_ = 544.4 ± 2.6	ε = 6.2 ± 0.2	161.5 ± 2.5 HV10	
AlSi10Mg/ 6.5 wt.%TiB_2_	BLT-S310 P = 260–350 W ν = 900–1500 mm/s d = 30 μm h = 110–170 μm	>99.5	1.63 μm for top	σ_y_ = 332.3 ± 6.7 σ_u_ = 536.9 ± 14.4	ε = 16.5 ± 1.7	-	[79]
			1.38 μm for side	σ_y_ = 277.9 ± 6.9 σ_u_ = 517.3 ± 9.1	ε = 15.4 ± 1.6		
AlSi10Mg/ 11.6 wt.% TiB_2_	House-built P = 200–300 W ν = 800–2000 mm/s d = 30 μm h = 105 μm E_v_ = 31.7–119.0 J/mm^3^	99.5	~2	σ_u_ = 530 ± 16	ε = 15.5 ± 1.2	191 ± 4 HV0.3	[80]
AlCu/ ~4.7 wt.% TiB_2_	Renishaw AM400 P = 250–300 W ν = 1125–4500 mm/s d = 30 μm h = 90 μm	Up to 99.5	0.5–2	σ_u_ = 391 ± 7.3 σ_y_ = 317.8 ± 9.3	ε = 12.5 ± 0.8	-	[50]
Al-Cu-Mg-Si/ 5 vol.% TiB_2_	SLM 250 HL P = 190 W ν = 165 mm/s d = 40 μm h = 80 μm E_v_ = 359.8 J/mm^3^	>99.0	2.5 ± 0.1	σ_yc_ = 191 ± 12	ε_c_ ≈ 60	-	[81]
Al-Cu/ ~4 wt.% TiB_2_	Aconity LAB P = 200 W ν = 1000 mm/s d = 30 μm h = 100 μm E_v_ = 66.67 J/mm^3^	99.9 ± 0.1	0.64 ± 0.26	σ_u_ = 401 ± 2	ε = 17.7 ± 0.8	113 ± 2 HV10	[82]
Al-12Si	SLM 250 HL P = 320 W ν = 1655 mm/s d = 50 µm h = 110 µm E_v_ = 35.1 J/mm^3^	-	-	σ_yc_ = 211 ± 4	-	119 HV0.05	[64,83]
Al-12Si/ 2 wt.% TiB_2_		≈99.1	~5.1	σ_yc_ = 225 ± 4	ε_c_ ≈ 30	142 ± 6 HV0.05	
AlSi10Mg	SLM125HL P = 300 W ν = 1650 mm/s d = 30 μm h = 130 μm E_v_ = 46.6 J/mm^3^ T = 200 °C	99.08 ± 0.1	6.1	σ_y_ = 243 ± 9 σ_u_ = 420 ± 9	ε_tr_ ≈ 5.5 ε_long_ ≈ 3.7	-	[84]
AlSi10Mg/ 0.05 wt.% LaB_6_		99.03 ± 0.08	4.0	σ_y_ ≈ 242 σ_u_ ≈ 430	ε_tr_ ≈ 6.4 ε_long_ ≈ 4.8		
AlSi10Mg/ 0.2 wt.% LaB_6_		99.17 ± 0.05	2.5	σ_y_ ≈ 245 σ_u_ ≈ 435	ε_tr_ ≈ 7 ε_long_ ≈ 6.5		
AlSi10Mg/ 0.5 wt.% LaB_6_		99.46 ± 0.18	2.2	σ_y_ ≈ 240 σ_u_ ≈ 427	ε_tr_ ≈ 6.5 ε_long_ ≈ 6.9		
AlSi10Mg/ 1 wt.% LaB_6_		99.49 ± 0.13	1.8	σ_y_ ≈ 235 σ_u_ ≈ 429	ε_tr_ ≈ 7.1 ε_long_ ≈ 5.8		
AlSi10Mg/ 2 wt.% LaB_6_		99.48 ± 0.22	1.6	σ_y_ ≈ 238 σ_u_ ≈ 445	ε_tr_ ≈ 7.0 ε_long_ ≈ 5.6		
2024 Al alloy	Aconity LAB machine P = 200–300 W ν = 600–1200 mm/s d = 30 µm h = 100 µm E_v_ = 56–167 J/mm^3^	98.3	-	-	-	66 ± 6 HV5	[28]
2024 Al alloy/ 2 wt.% CaB_6_		>99.5	0.91 ± 0.32	σ_y_ = 348 ± 16 σ_u_ = 391 ± 22	ε = 12.6 ± 0.6	132 ± 4 HV5	

*E_v_—laser volumetric energy density, E_l_—laser linear energy density, P—laser power, ν—scanning speed, h—hatching distance, d—layer thickness, σ_u_—ultimate tensile strength, σ_y_—yield strength, σ_uc_—ultimate compressive strength,σ_yc_—compressive yield strength, ε—elongation, ε_long_—elongation at longitudinal direction, ε_tr_—elongation at transverse direction, ε_c_—compression strain, RT—room temperature, - means no data available*.

A microstructure with average grain size of 1.38 µm for the vertical sector was observed [79] when 6.5 wt.% TiB_2_ was added (Figure 3k). However, the increase in TiB_2_ content to ~11.6 wt.% (almost two times) [80] did not result in further grain refinement (Figure 3l).

Partial melting of TiB_2_ was reported in Ref. [73] despite the fact that TiB_2_ is considered a refractory material. Adding 5 vol.% (or 8.3 wt.%) TiB_2_ to an Al–Cu alloy [81] resulted in a remarkable grain size reduction from 23 to 2.5 µm. In Ref. [82], the in situ TiB_2_ (4 wt.%) reinforced Al–Cu–Ag–Mg–Ti alloy had fine equiaxed grains with ~0.64 µm average size without preferential orientation (Figure 3p). The reported grain size was smaller than that stated in Refs. [73,80]. In Refs. [64,83], the addition of 2 wt.% TiB_2_ to an Al–12Si alloy produced a textureless microstructure with an average grain size of ~5 µm, meaning that in case of similar content of incorporated TiB_2_, coarser grains were grown in the Al–12Si alloy than in AlSi10Mg (Figure 3m,n). For comparison, a hot-pressed sample’s EBSD image is shown in Figure 3o, which, interestingly, showed a higher degree of grain refinement.

For a bare minimum boride additive range, at least 2 wt.% TiB_2_ is sufficient to significantly alter the final morphology and crystallographic texture of LPBF-processed materials [64,73,77,82,83].

**Figure 4:**
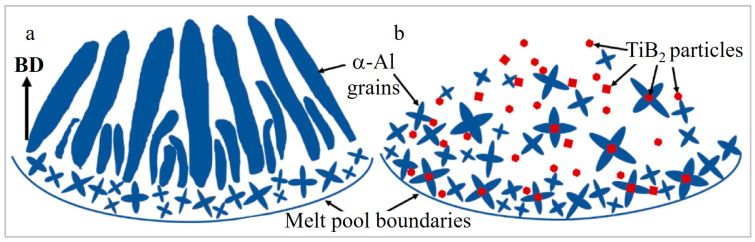
Graphical illustration of grain formation during solidification in a melting pool of AlSi10Mg (**a**) and AlSi10Mg-TiB_2_ AMC (**b**) (reproduced with permission from [77]).

The grain refining (columnar to equiaxed transition) effect of TiB_2_ (Figure 4a,b) is ascribed to its good stability in a melt pool, supplying numerous low-energy barrier nucleation sites (crystal embryos) and a reduction in the critical amount of total undercooling required to initiate the formation of equiaxed crystals [77]. The particles pushed to the grain boundaries pin and stabilize grain boundaries and limit grain growth along the heat flux direction [59]. Furthermore, due to a lower thermal conductivity of TiB_2_ (~77.8 W/mK) as compared to Al (~108 W/mK) [73], TiB_2_ particles prevent heat flux at a high temperature, reducing the temperature gradient. The latter results in the formation of fine equiaxed grains, weakening the texture and anisotropy of fabricated AMCs [59]. Overall, grain refinement is justified with a combination of high cooling rates during LPBF, an increased number of nucleation sites and limitations on grain growth [73,80], which lie beneath three main mechanisms: constitutional supercooling, heterogeneous nucleation and Zener pinning. Meanwhile, random orientations of TiB_2_ particles provide the randomization of Al grain orientation and texture elimination [77].

The grain refining effect of TiB_2_ is also reported to be a result of the formation of Al_3_Ti and the crystallographically coherent interface between Al_3_Ti and TiB_2_, which promotes the nucleation of Al_3_Ti on the surface of TiB_2_ particles in an Al melt. Without the Al_3_Ti layer, TiB_2_ additives are easily contaminated by impurities with a high tendency to form a eutectic microstructure with Al and, therefore, being insufficient in nucleating α-Al grains [85]. However, in Ref. [81], a preferable natural stacking sequence of Al atoms on TiB_2_ and direct refining are reported. Meanwhile, in Ref. [82], it was highlighted that the absence of the Al_3_Ti layer does not prove a lack of nucleation, since the Al_3_Ti layer can fully transform into α-Al during the cooling process via a peritectic reaction.

**Figure 5:**
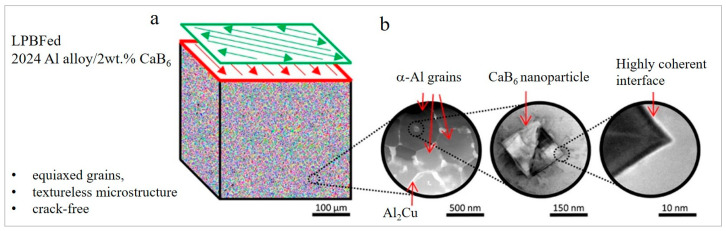
EBSD inverse pole figure grain orientation map of LPBFed 2024 alloy—2 wt.% CaB_6_ (**a**), respective HAADF-STEM and ADF-STEM images of CaB_6_ nanoparticles within α-Al grain (**b**) (HAADF-STEM stands for high-angle annular dark-field scanning transmission electron microscope, ADF for annular dark-field)(reproduced with permission from [28]).

Besides TiB_2_, other borides, such as CaB_6_ and LaB_6,_ had shown a promising refining capability. The addition of 0.05–2 wt.% LaB_6_ to AlSi10Mg resulted in grain refinement down to 1.6 µm (Figure 3q–t). LaB_6_ particles form a highly coherent interface with the Al matrix. A higher amount of LaB_6_ nanoparticles (>0.5 wt.%) did not further provide grain refinement and restricted longitudinal elongation due to the weakening of melt pool boundaries by segregation of the excess LaB_6_ nanoparticles [84]. The addition of 2 wt.% CaB_6_ nanoparticles to the high-strength 2024 aluminum alloy resulted in an equiaxed, crack-free microstructure with an average grain size of 0.91 ± 0.32 µm and a highly coherent interface with Al (Figure 3u,v and Figure 5a,b) [28]. No decomposition of CaB_6_ was observed. However, not every CaB_6_ nanoparticle functions as a nucleant; a large quantity of them is acquired in the liquid phase between the growing grains, and they are forced to the grain boundaries where they stabilize the microstructure via Zener pinning.

In Ref. [77], the addition of 0.5–8 wt.% TiB_2_ to AlSi10Mg resulted in increased strength (up 544 MPa) and hardness (with 20%); however, the high content of TiB_2_ (>2%) resulted in a reduced ductility (6.2%), which was still higher than for a reference AlSi10Mg. Simultaneous enhancement of strength (up to 537 MPa and 530 MPa) and ductility (16.5% and 15.5%) was achieved in Refs. [79,80], respectively, when 6.5 wt.% and 11.6 wt.% TiB_2_ were introduced to AlSi10Mg. The increased strength was mainly attributed to the Hall–Petch relationship, loading-bearing and Orowan strengthening mechanisms. The grain boundary modification by TiB_2_ nano-particulates and the promoted dislocation plasticity by nano-Si precipitates improved ductility. LaB_6_ addition resulted in a subtle improvement of strength and ductility; however, the reinforcing effect was not as pronounced, as in the case of TiB_2_.

The highest elongation (~17.7%) was recorded in Ref. [82], when the Al–Cu alloy was reinforced with 4 wt.% TiB_2_; however, the alloys exhibited a significantly lower strength and hardness. The addition of 2 wt.% CaB_6_ [28] resulted in an increased elongation of 2024 alloy, up to 12.6%, and improved tensile and yield strength (Table 1).

### 2.2. Carbides: Grain Refining and Strengthening Effect of TiC, SiC, B_4_C

#### 2.2.1. Titanium Carbide: TiC

TiC exhibits several favorable characteristics required for Al alloys reinforcement; among them, there are moderate density (4.91 g/cm^3^), high hardness (28–32 GPa) [86], high modulus of elasticity (up to 440 GPa) [87], good wettability, good laser absorptivity (higher than TiB_2_) and low lattice mismatch (6.9%) with Al. TiC particle reinforced AMCs have a high strength, stiffness and modulus, good corrosion and wear performance [22,72]. However, when formed in situ in the melt pool, the TiC phase possesses unstable chemical composition (portrayed as TiC_x_, where x is in 0.48–1 range) due to the generation of carbon atom vacancies. Consequently, the nucleating behavior of TiC_x_ for α-Al is not consistent, since the TiC_x_+Al→Al_4_C_3_ reaction is favored, which results in weakened grain refining performance [88].

**Figure 6:**
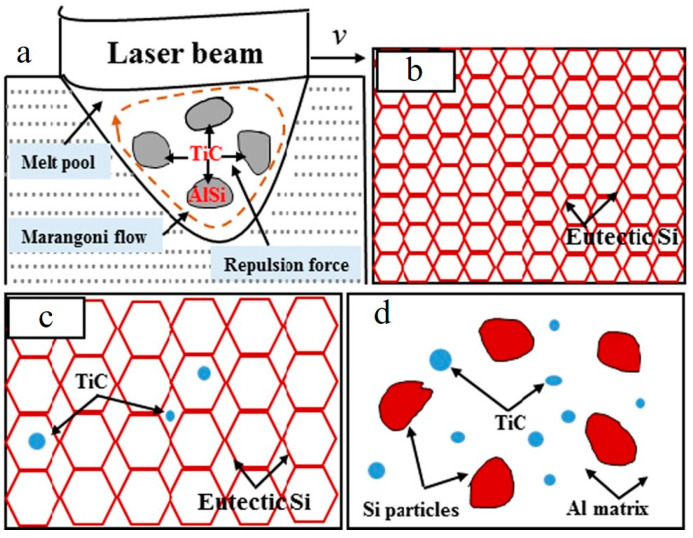
Microstructure evolution of the Al–15Si alloy reinforced with 1 wt.% (**a**), 2.5 wt.% (**b**), 7.5 wt.% (**c**) and 10 wt.% TiC (**d**) (reproduced with permission from [89]).

In Ref. [89], an increase in the TiC content from 1 to 10 wt.% when added to the Al–15Si alloy resulted in an increase in melt pool fluidity and a decrease in the undercooling degree, leading to significant grain coarsening (Figure 6). Ultimately, with the added threshold limit of TiC (10 wt.%), the primary Si particles precipitate out and distribute on the surface of the Al matrix (Figure 6d).

**Figure 7:**
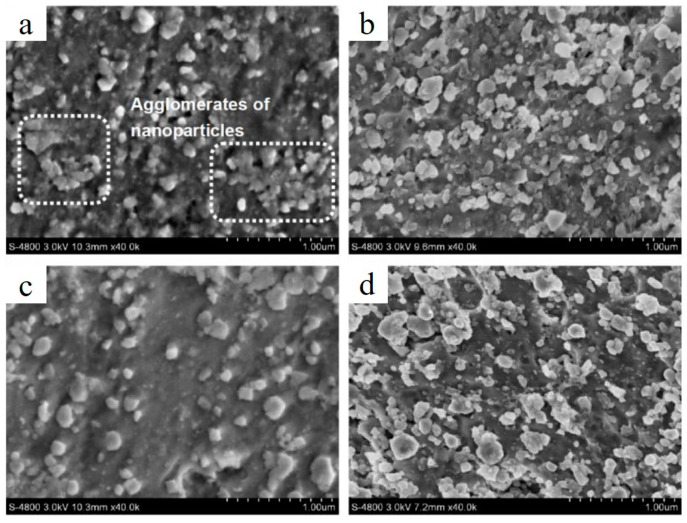
SEM images portraying dispersion degree of TiC and respective microstructure of fabricated AlSi10Mg/5 wt.%TiC composite processed at various E_l_ (E_v_): 314 J/m (125.71 J/mm^3^) (**a**), 440 J/m (176.0 J/mm^3^) (**b**), 733 J/m (293.3 J/mm^3^) (**c**) and 1100 J/m (440.0 J/mm^3^) (**d**) (reproduced with permission from [70]).

Alternatively, the fabrication of AlSi10Mg/5 wt.%-nano-TiC [70] under an increased laser energy caused the nano-TiC particles to accumulate in clusters, forming the micron-sized agglomerates. However, the dispersion of reinforcement became more uniform, as shown in Figure 7a–d.

**Figure 8:**
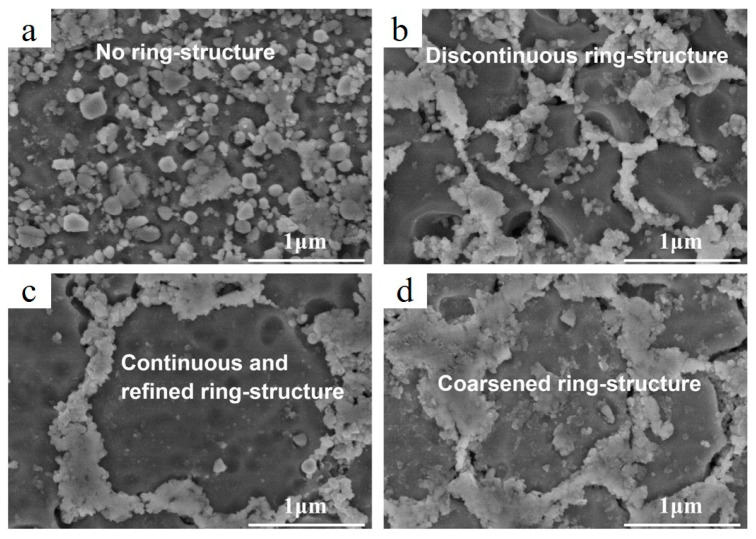
SEM images demonstrating the dispersion states of nano-TiC particles in LPBFed AlSi10Mg/3 wt.%TiC composites at E_v_ = 160 J/mm^3^ (**a**), E_v_ = 200 J/mm^3^ (**b**), E_v_ = 240 J/mm^3^ (**c**) and E_v_ = 280 J/mm^3^ (**d**) (reproduced with permission from [71]).

An increase in energy input resulted in change in TiC appearance, from aggregate to ring (circular) structures, due to intensive Marangoni flow (Figure 8a–d) in LPBFed AlSi10Mg/3 wt.%TiC composites [71].

**Figure 9:**
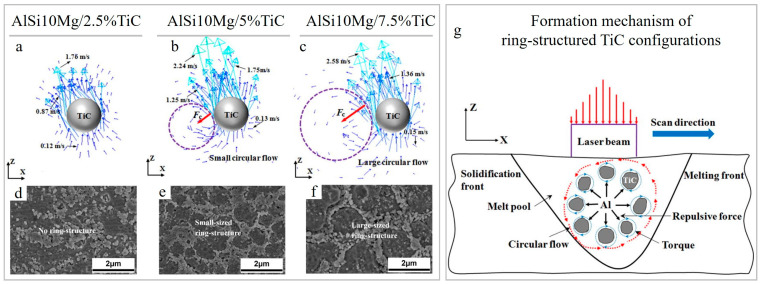
Velocity vector plots around a TiC reinforcing particle in the melt pool (the dashed circles highlight the circular motion) and micrographs demonstrating typical morphology of LPBF-processed AlSi10Mg/TiC nanocomposites with different TiC contents: 2.5 wt.% (**a**,**d**), 5 wt.% (**b**,**e**) and 7.5 wt.% (**c**,**f**). Schematics of the formation mechanism of novel circular TiC configurations during fusion process at fixed E_v_ = 571.43 J/mm^3^ (**g**) (reproduced with permission from [22]).

The formation of ring-structured TiC was reported in Ref. [22] as well. At 5 and 7.5 wt.% TiC addition, at elevated Marangoni force and a lower viscous drag force, the ceramic particulates are captured in the circular melt motion (Figure 9b,c) and generate distinct circular structures in solidified build (Figure 9e–g). The circular-structured TiC agglomerate formation was not found in Ref. [70] when 5 wt.% TiC was used, which can probably be justified by the application of different process parameters.

**Figure 10:**
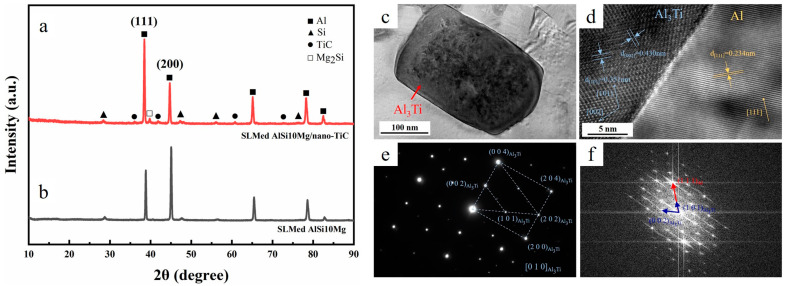
Diffractograms of the LPBFed AlSi10Mg (**a**) and AlSi10Mg/5 wt.%TiC (**b**) specimens, HRTEM image of the D0_22_–Al_3_Ti/Al matrix (**c**) and interface (**d**), SAED patterns taken at the D0_22_-Al_3_Ti along (010) Al_3_Ti (**e**), FFT patterns of the D0_22_–Al_3_Ti/Al matrix interface (**f**) (SAED stands for selected area electron diffraction and FFT for fast Fourier transform, (reproduced with permission from [31]).

**Figure 11:**
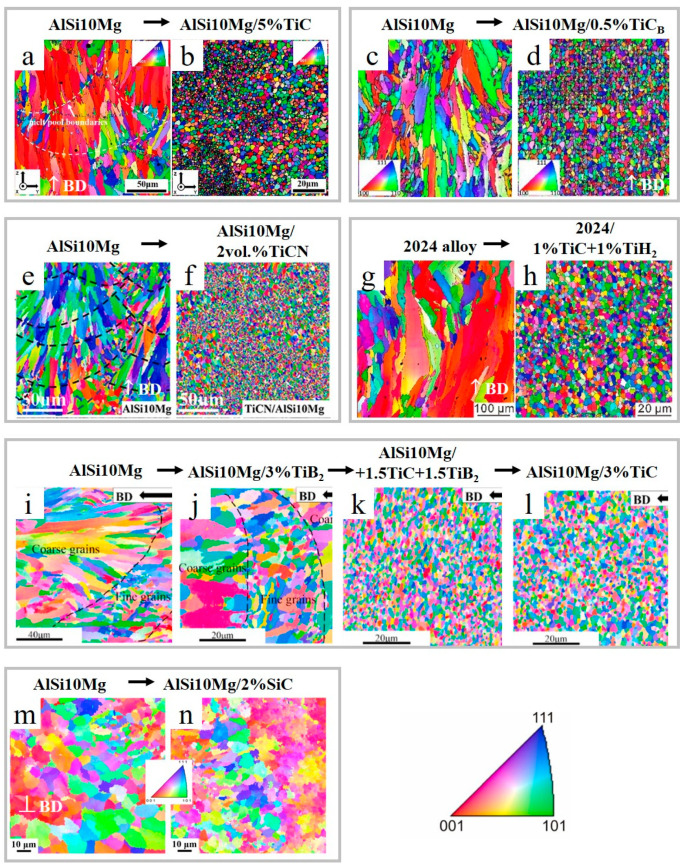
EBSD color maps for LPBF-prepared Al alloys and AMCs reinforced with carbides, carbonitride, carbide/hydride and carbide/boride additives (**a**–**n**) (reproduced with permission from [31,88,91,92,93]).

The presence of in situ formed D0_22_-Al_3_Ti inoculants (with tetragonal structure) was revealed in Ref. [31] for the AlSi10Mg/5 wt.%TiC composite. Heterogeneous nucleation of α-Al on the D0_22_-Al_3_Ti nanoparticles (Figure 10c–f) occurred, leading to (i) columnar-to-equiaxed transition with subsequent grain refinement from ~80 µm to ~1 µm (Figure 11a,b), and (ii) the preferred orientation of the of α-Al (200) phase was removed (Figure 10a,b). In situ formed Al_3_Ti served as a more effective nucleant as compared to TiC, mainly due to the small lattice mismatch between Al and Al_3_Ti, which was reduced to 0.09%.

Yet, another variable parameter centers on powder production for the LPBF process. In Ref. [90], the LPBF of the ball-milled composite powder of AlSi10Mg/5 wt.%TiC is reported. After printing, the TiC particles maintained their nanoscale nature and were not subjected to a significant coarsening, which resulted in an increased hardness of the alloy from 140 to 185 HV_0.1_ and the tensile strength from 400 to 482 MPa (Table 2). The elongation of the composite part (10.8%) was similar to the elongation measured for the pure AlSi10Mg alloy. This can be explained by various effects: (i) an increased dislocation density near reinforcement/matrix interface, (ii) TiC nanoparticles acting as a barrier for dislocation movement, (iii) delaying crack propagation, thus improving the tensile strength. Alternating the TiC concentration, laser energy density and powder processing technique yield different composite attributes, as shown in Table 2.

**Figure 12:**
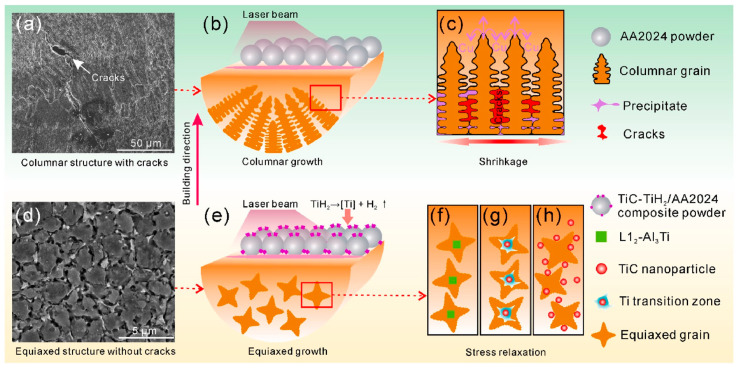
Schematic representation of microstructures and solidification mechanisms of LPBF-fabricated 2024 Al alloy (**a**–**c**) and 2024/TiC-TiH_2_ composite (**d**–**h**) (reproduced with permission from [92]).

While using a single carbide reinforcement has proven to be an effective way for grain refinement, the use of a second additive was shown to complement the effects of a single species. In Ref. [92], the dual reinforcing phases were used, resulting in a crack-free sample produced from the 2024 alloy/1 wt.%TiC+1 wt.%TiH_2_ powders mixture. It was shown that unreinforced alloy contained columnar microstructure (Figure 11g and Figure 12a–c), while the 2024 alloy/1 wt.%TiC+1 wt.%TiH_2_ composite was composed of superfine equiaxed grains (Figure 11h and Figure 12d–h).

**Figure 13:**
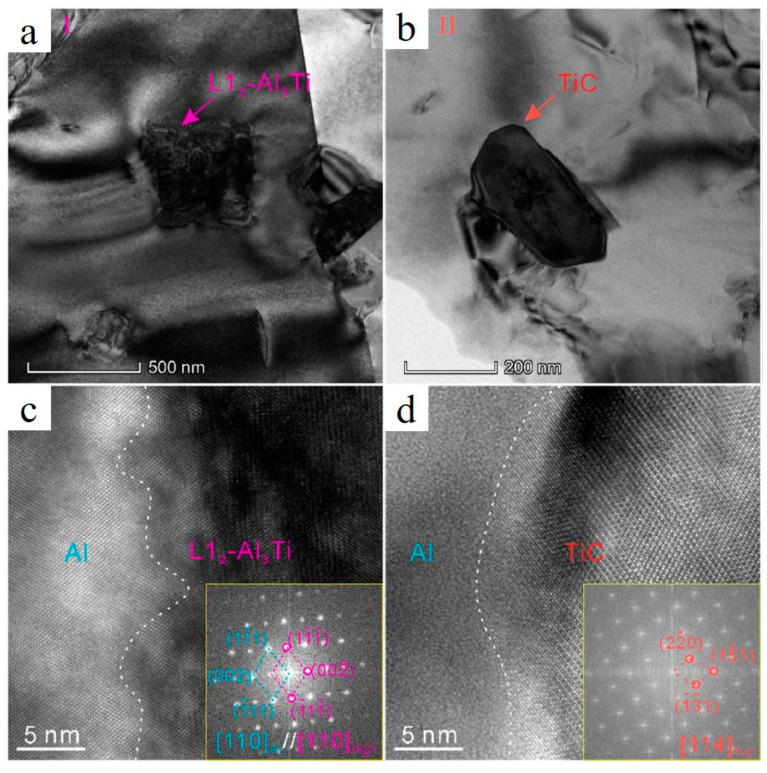
TEM images of L1_2_-Al_3_Ti (**a**) and TiC particles (**b**), HRTEM image and respective FFT pattern of α-Al/L1_2_-Al_3_Ti interface (**c**) and α-Al/TiC interface (**d**) (reproduced with permission from [92]).

Ti-rich particles (TiC and Al_3_Ti) with irregular or cubic shape are present in the grains exhibited in Figure 13a,b. The L1_2_-Al_3_Ti with a face-center-cubic (FCC) structure is a result of TiH_2_ decomposition (TiH_2_→Ti+H_2_) and reaction between Ti and Al melt. It is worth mentioning that in Ref. [31], a formation of the D0_22_-Al_3_Ti phase with a tetragonal structure was reported. A highly coherent interface between L1_2_-Al_3_Ti and α-Al was observed (with 0.24% lattice mismatch) (Figure 13c), indicating that L1_2_-Al_3_Ti might serve as substrate for heterogeneous α-Al nucleation; however, a coherent interface was not generated between TiC and Al (Figure 13d). Following the “Ti transition zone” theory (demonstrated in Figure 12), Ti-covered TiC nanoparticles, and then TiC particles themselves, become the effective nucleation substrates for α-Al as well.

On account of the inhibition of columnar grains, elimination of cracks, a refined microstructure and Orowan strengthening, the 2024 alloy/TiC+TiH_2_ AMC showed a simultaneous enhancement of tensile strength and ductility.

Another study on the fabrication of double TiB_2_-TiC reinforced AMCs [93] revealed that the addition of dual ceramic phases improved laser absorptivity by almost two-fold, substantially refining the Al grains (Figure 11i,k) and resulting in the increment in tensile strength (552 MPa) and elongation (12%) (Table 2). It was revealed that the dual reinforcement more remarkably affected the mechanical performance, improved densification and grain refinement compared to the single reinforcement with the same total content (Table 2 and Figure 11j,l).

**Figure 14:**
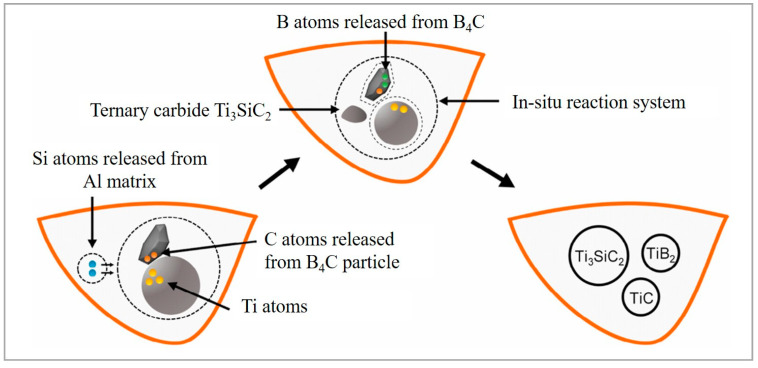
In situ formation mechanism of TiB_2_, TiC, Ti_3_SiC_2_ ceramic phases in the molten pool (reproduced with permission from [94]).

Double or triple reinforcements formed during in situ chemical reactions generate a composite material highly coherent with the metal matrix. When 0–17.2 wt.% (Ti-B_4_C) mixture was added to AlSi10Mg [94], the full densification of samples and in situ formation of ceramic phases were reported due to the combined LPBF and combustion synthesis (CS) process. Silicon atoms released from the alloy combine with Ti and C atoms, yielding the formation of transitional ternary carbide Ti_3_SiC_2_, while the remaining B_4_C and Ti are responsible for the formation of TiB_2_ and TiC particulates (Figure 14). The generation of the Ti_3_SiC_2_ phase resulted in a significant drop in porosity of the fabricated sample. The heat released during the combustion reaction allowed for carrying out the fabrication in low laser energy regime.

#### 2.2.2. Silicon Carbide: SiC

The SiC particle reinforced AMCs are applied in aerospace and electronic encapsulation, both in military and civilian fields, due to their high specific strength and stiffness, in addition to abrasion resistance. SiC has a much higher laser absorptivity (≈78%) than aluminum (≈7%), moderate density (3.21 g/cm^3^), and it increases the laser absorptivity of the blended mixture [13,34,97,98]. During laser irradiation, SiC particles tend to heat up to extremely high temperature, leading to rapid reaction rates. Hence, the decrease in thermal conductivity results in further rise in temperature, the lifetime and fluidity of the melt pool. Meanwhile, an increase in SiC content in the initial feedstock and, hence, in the blend melt pool, increases the viscosity of a liquid melt and results in a lower fluidity. Therefore, both thermo-kinetic factors should be considered before selecting the content and size of the reinforcing SiC [11,13].

The chemical reaction between silicon carbide and aluminum melt at temperatures exceeding 940 K may result in SiC decomposition according to 4Al(l)+3SiC(s)→Al_4_C_3_(s)+3Si(s) reaction. Al_4_C_3_ compound is known to be brittle and unstable, causing degradation of the mechanical properties of the AMCs. It is reactive with H_2_O in humid conditions and might form amorphous aluminum hydroxide. This process is followed by a volume increase and can induce the residual stresses into the surrounding aluminum matrix. Therefore, the inhibition of the Al_4_C_3_ formation is a crucial issue to be overcome [11,34].

At a processing temperature above 1670 K, Al_4_SiC_4_ (ternary carbide) is formed following the 4Al_(l)_+4SiC_(S)_→Al_4_SiC_4(S)_+3Si reaction [13]. Al_4_SiC_4_, due to its high hardness of 1200 HV, low brittleness, remarkable chemical stability in wet conditions, is a favored reinforcement for aluminum [11]. At temperatures above 2800 °C, SiC particles partially or fully decompose into silicon and carbon vapor [34,97]. The increase in applied energy results in a high degree of SiC decomposition, causing surface turbulence, melt pool instability, non-continuous scan tracks and, consequently, an uneven surface finish.

**Figure 15:**
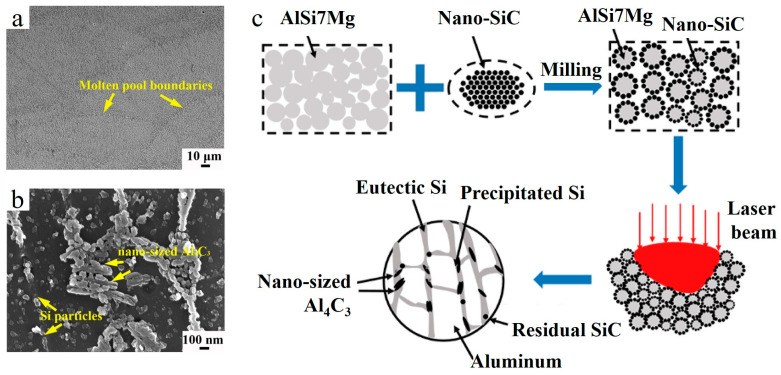
Cross section SEM images of the LPBF-ed AlSi7Mg/2 wt.% nano-SiC composite (**a**,**b**) and the illustration of the formation route of different phases during the LPBF process (**c**) (reproduced with permission from [8]).

It should be noted that the size of used SiC reinforcing particles ranges from tens of micrometers down to nanoscale, and the resultant mechanical properties of AMCs are significantly affected by particle size [8,13]. In Refs. [8,34], the LPBF of AlSi7Mg/2 wt.% nano-SiCp (40 nm) and Al-12Si/10 vol.%SiC (≈11.7 wt.%) (SiC ≈ 25 μm), respectively, were reported. Nano SiC in AlSi7Mg matrix serves as a grain refinement agent (Figure 11m,n) due to the nucleation of numerous heterogenous sites and formation of nanosized Al_4_C_3_ (Figure 15b,c). The use of nano-SiC yielded low porosity, near-full densification and improvement in tensile strength without sacrificing ductility. However, inferior densification was observed in Ref. [34] when a micron size reinforcement was used.

**Figure 16:**
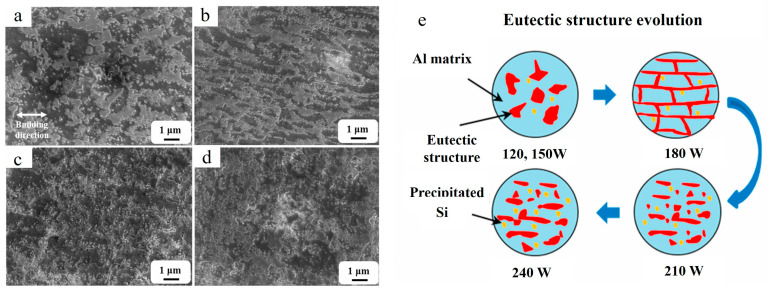
High-magnification SEM micrographs of as-built AlSi10Mg/SiC composites fabricated at different laser powers of 120 W (**a**), 180 W (**b**), 210 W (**c**), 240 W (**d**) and graphical illustration for development of eutectic structure (**e**) (reproduced with permission from [95]).

The successful fabrication of AlSi10Mg/2 vol.% nano-SiC (~2.4 wt.%) composite reinforced by Al_4_SiC_4_ phase was reported in Ref. [95]. With an increase in laser power, the eutectic structure gradually changed from thick flakes to network shapes and then to a fine structure, as shown in Figure 16.

**Figure 17:**
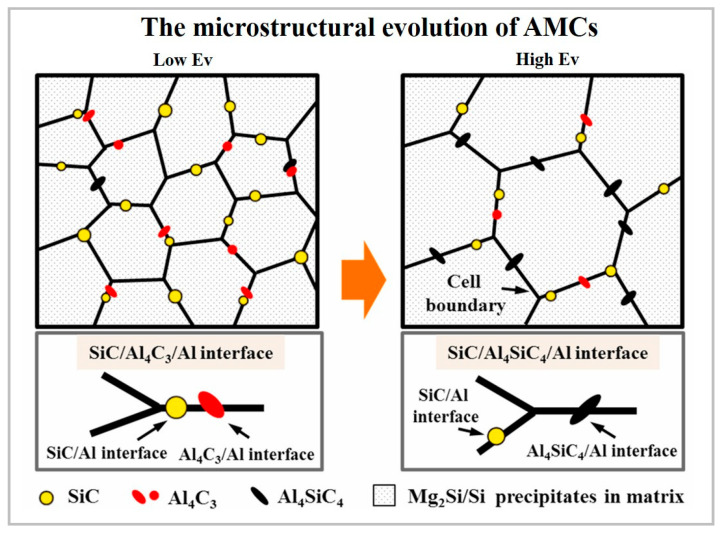
Microstructure changes of the composites at low to high energy application (reproduced with permission from [95]).

At low applied energy, the eutectic structure represents a collection of thick flakes. In contrast, high energy input provides sufficient wettability between SiC and Al, promoting the reaction product transformation into Al_4_SiC_4_ and a homogeneously dispersed eutectic structure (Figure 17), which positively affects the mechanical properties of the AMC. Despite the analogous content of nano SiC added to the Al alloy, the mechanical properties of the samples in this work are far inferior to those reported in Ref. [8].

An increase in SiC content up to 10 wt.% resulted in increased tensile and yield strength; however, the SiC, Si and in situ formed Al_4_SiC_4_, reduce the elongation of the composites [96]. When comparing the properties of AlSi10Mg/15–20 wt.% SiC composites [11,13,97,98], it should be mentioned that the highest hardness (316.2HV_0.2_) and density (98.9%) were achieved for AlSi10Mg/15 wt.%SiC, when the SiC particle size was 1200 mesh [98] (Table 2). The larger SiC particles reduced tensile strength as compared to a pure alloy [97]. The use of finer SiC particulates yields to a higher degree of densification, elevated microstructural uniformity and simultaneous improvement in compressive strength, hardness and strain [11,98]. In Refs. [11,13], the in situ formed Al_4_SiC_4_ is shown to serve as a transition zone, limiting the interaction of SiC and aluminum crystals simultaneously with reinforcing capacity for the Al.

### 2.3. Nitrides: Grain Refinement and Strengthening Effect

#### 2.3.1. Titanium Nitride: TiN

Besides the favorable characteristics of ceramic materials, TiN (titanium nitride) also demonstrates excellent light absorptivity. TiN has good coherency with Al, owing to small difference (4.72%) in lattice parameters (a_Al_ = 0.4049 nm and a_TiN_ = 0.4240 nm). Meanwhile, the laser reflectivity (at 1064 nm laser wavelength) of the AlSi10Mg/TiN composite powder is around 25%, which is much lower than that of AlSi10Mg powder (62%) [99].

**Figure 18:**
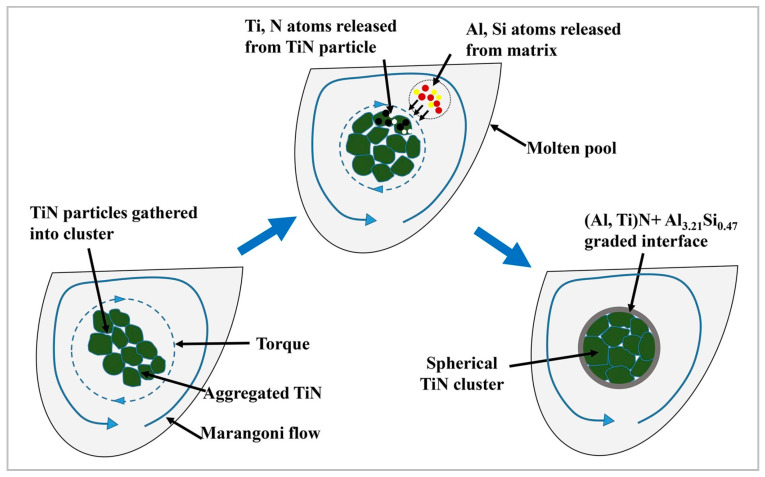
Graphical representation of the movements of aggregated TiN particles and the novel graded layer formation mechanism (reproduced with permission from [100]).

In Refs. [99,100], when fabricating AlSi10Mg/2 wt.%TiN composite, the mutual diffusion and in situ reaction between the TiN clusters and aluminum generates a graded interfacial layer composed of Al_3.21_Si_0.47_ and (Ti,Al)N (Figure 18).

The formed layer is of central importance to the enhancement in microhardness due to an improved interface bonding and a precipitation of stiff (Al,Ti)N. The combined influence of superfine grains (0.284 µm), uniform particle dispersion, formed novel layer and high densification significantly improve the mechanical and wear characteristics of the fabricated AMCs. The Al matrix–Mg_2_Si–TiN coherent interfaces lead to a precipitation strengthening, benefiting the enhancement in strength [100].

**Figure 19:**
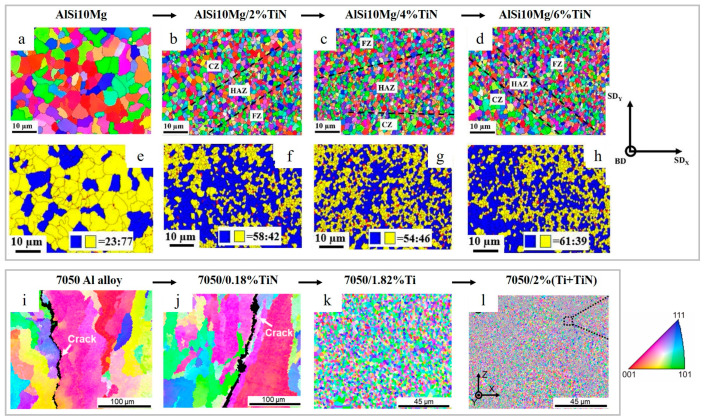
EBSD orientation maps from the top view and distribution of sub-structured (in yellow) and recrystallized (in blue) grains of the as-built AlSi10Mg reinforced with 0% TiN (**a**,**e**), 2%TiN (**b**,**f**), 4% TiN (**c**,**g**) and 6%TiN (**d**,**h**) EBSD color maps of 7050 Al alloy (**i**), 7050-0.18TiN (**j**), 7050-1.82Ti (**k**) and 7050-2(Ti+TiN) (**l**) (reproduced with permission from [66,101]).

**Figure 20:**
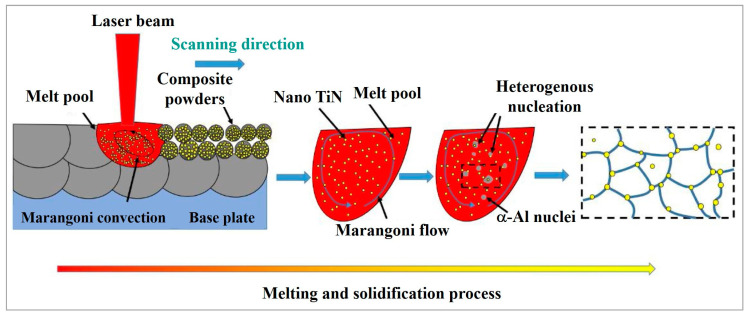
Graphical illustration demonstrating the morphology evolution for the TiN/AlSi10Mg AMC during LPBF (reproduced with permission from [101]).

An increase in TiN content (0–6 wt.%) improves strength, ductility and hardness of nano-TiN particle reinforced AlSi10Mg [101]. It was shown that 4 wt.% TiN is a critical threshold to inhibit porosity. The composites had a relatively random grain orientation, and the grain size decreased from 3.86 to 1.19 μm when the content of TiN increased from 0–6 wt.% due to intensive heterogenous nucleation (Figure 19a–d and Figure 20, Table 3).

As shown in Figure 20, only a fraction of TiN serves as heterogenous nucleation substrates, and the majority of particles are dispersed along the grain boundaries owing to the pushing effects of the solidification front.

It was found that all the specimens were dominated by high-angle grain boundaries (HAGBs), and with an increase in TiN content, the volume of low-energy HAGBs increased. TiN nanoparticles also promote recrystallization and possesses a crucial role in recrystallized nucleation during the LPBF process, as shown in Figure 19e–h.

**Figure 21:**
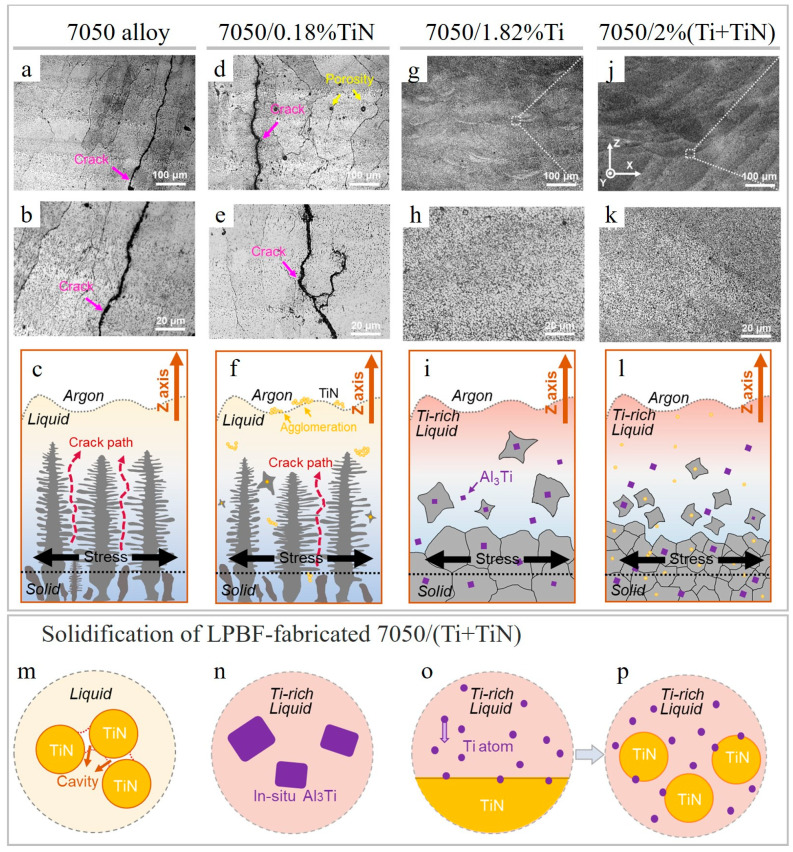
SEM images of LPBF-fabricated 7050 alloy (**a**,**b**), 7050/0.18%TiN (**d**,**e**), 7050/1.82%Ti (**g**,**h**) and 7050/2%(Ti+TiN) (**j**,**k**) samples after etching. Schematic diagram of solidification, columnar and equiaxed grain formation of fabricated 7050 (**c**), 7050-TiN (**f**), 7050-Ti (**i**) and 7050/(Ti+TiN) (**l**). Solidification of 7050/(Ti+TiN): agglomeration of TiN particles in high-temperature liquid Al (**m**), in situ Al_3_Ti in Ti-rich liquid Al (**n**), Ti absorption at the interface between TiN and liquid Al (**o**), dispersion of TiN in Ti-rich liquid Al (**p**) (reproduced with permission from [66]).

The use of hybrid Ti–TiN reinforcements for 7050 Al alloy was reported in Ref. [66], exhibiting significant synergistic grain refinement and a higher strengthening as compared to pure 7050 Al alloy and a single reinforced 7050-TiN and 7050-Ti. Although both single-Ti-reinforced and hybrid-reinforced alloy possessed a crack-free microstructure (Figure 21g–l), the hybrid reinforcement provided greater grain refinement (Figure 19k,l).

Meanwhile, the 7050 and 7050-0.18%TiN specimens are prone to cracking, consist of columnar grains and possess relatively high porosity (Figure 19i,j and Figure 21a–f). The reason for grain refinement, when Ti is added to pure alloy and to 7050-TiN, is the formation of L1_2_ structured Al_3_Ti, which promotes heterogeneous nucleation and contributes to the rapid formation of constitutional supercooling zones (Figure 21n). Besides Al_3_Ti, fine MgZn_2_ phase was formed with coherent interface with Al; however, the in situ formed Al_2_CuMg showed non-coherent interface with Al. Ultrafine grains (775 nm) were reported in the LPBF-prepared 7050-2 wt.%(Ti+TiN) composite, vastly benefiting from the Ti/TiN synergism.

It can be concluded that the addition of 2–4 wt.% TiN-Ti hybrid additives notably improved the quality of LPBF-fabricated AMCs.

#### 2.3.2. Aluminum Nitride: AlN

AlN is one of the favorable reinforcing candidates for aluminum alloys due to its superior combination of high thermal conductivity (~250 W/mK) [105] and high hardness (~12 GPa) [106]. AlN shows high chemical stability, good compatibility with Al alloy combined with a good interfacial adherence without any interfacial reaction [107]. Besides, due to a low thermal expansion coefficient (similar to Si), AlN has been broadly employed in the aviation and transportation and is shown to be an appropriate reinforcement for aluminum alloys [102].

**Figure 22:**
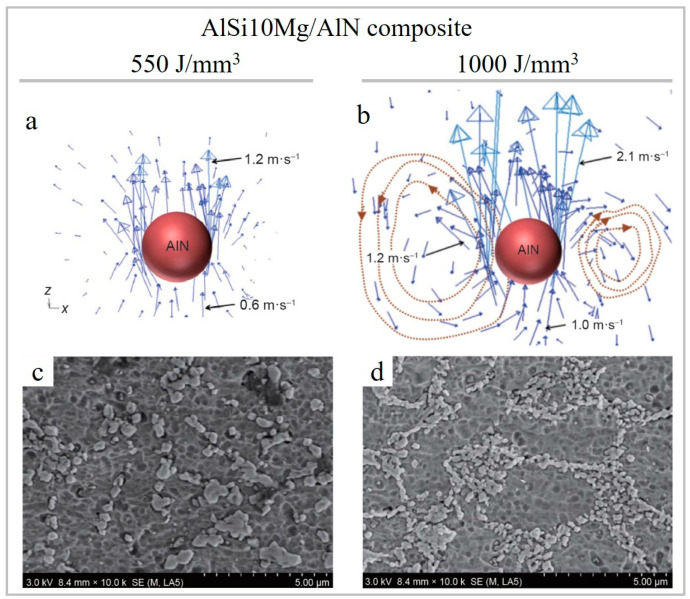
Characteristics of velocity vector obtained around AlN reinforcing particles and their respective distribution state in the solidified Al matrix at E_v_ = 550 J/mm^3^ (**a**,**c**) and E_v_ = 1000 J/mm^3^ (**b**,**d**) (reproduced with permission from [108]).

In a series of works [67,107,108], it was observed that the applied energy had a dramatic effect on the AlN particle distribution. At low energy, random AlN distribution occurred due to the relatively consistent pressure around the introduced particles (Figure 22a,c); and at high laser energy, a circular-structured AlN distribution was compelled by the centripetal force (Figure 22b,d).

However, excessive energy results in particles coarsening and a deconstruction of the circular-structured AlN. In Ref. [58], the preparation of an almost fully densified composite with 1 wt.% AlN and refined grains of increased wear resistance has been reported. In Ref. [102], it was shown that during LPBF of AlSi10Mg-2 wt.%AlN powders mixture, the solidified material undergoes various microstructural transformations from the first to the fourth layer (directional columnar microstructure to coarse cellular microstructure), affirming the importance of added particles, solidification rate, the lifespan of the melt pools and subsequent crystal growth rate.

#### 2.3.3. Boron Nitride: BN

The high tensile strength and low density (2.1 g/cm^3^, which is close to that of pristine Al), makes hexagonal boron nitride (h-BN) an effective reinforcing agent for the AMCs [109]. It was revealed that even 1 wt.% addition of BN micro-flakes to AlSi10Mg increased the tensile strength and hardness as compared to a pure alloy due to the formation of AlN and AlB_2_ phases via solid-state Al–BN reaction [103].

#### 2.3.4. Silicon Nitride: Si_3_N_4_

A whole basket of favorable properties of Si_3_N_4_ (silicon nitride), including remarkable strength, high hardness, high elastic modulus, lower CTE, superior hardness compared to other ceramics, [110,111,112], similar density with aluminum, which will ensure homogeneous dispersion, and high wettability with the aluminum matrix [104] makes it a promising reinforcing agent. The enhanced strength and elastic modulus of the LPBF-prepared AlSi10Mg-Si_3_N_4_ composite, owing to the impeded dislocation motion during deformation and load-bearing effect of added reinforcing Si_3_N_4,_ are achieved. The mutual diffusion of Al and Si atoms and the absence of in situ formed brittle phases increased the Al matrix-Si_3_N_4_ particles bonding strength [104]. The addition of Si_3_N_4_ to the Al alloy, however, reduces process stability and thus narrows the optimal range of process parameters [104].

## 3. Comparison of Ceramic Reinforcements’ Influence on LPBF Process and the Properties of the AMCs

As shown above, even small portions of ceramic or hybrid additives (metal–ceramic), such as 0.5–0.7 wt.%, are able to dramatically improve the performance of the AMCs. Accordingly, matching ceramic additives with an optimized fraction and particle size provides good wettability, compatible interfaces and a strong bonding between the constituents, which hinder crack propagation and contribute to a hardening and strengthening of AMCs.

**Figure 23:**
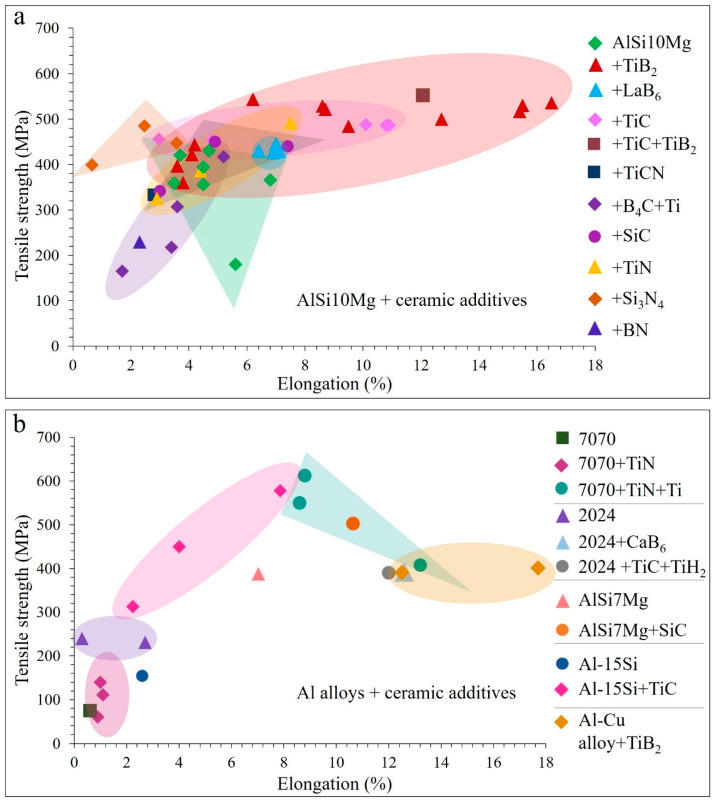
Tensile strength and elongation results of LPBF prepared ceramic particulate reinforced AlSi10Mg (**a**) and other Al alloys (**b**) (data from [8,28,31,50,59,66,71,73,77,79,80,82,84,88,89,90,91,92,93,94,95,96,97,98,101,103,104]).

**Figure 24:**
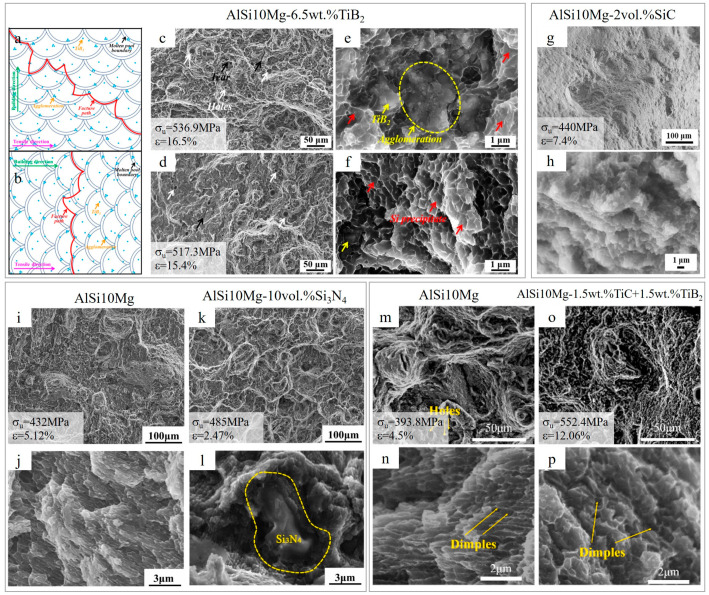
The schematic diagram of probable crack propagation path (**a**,**b**) and tensile fracture morphology for horizontal (**c**,**e**) and vertical samples (**d**,**f**) of the AlSi10Mg/6.5 wt.%TiB_2_ composite. Fracture SEM images of AlS10Mg/2 vol.%SiC (**g**,**h**), AlSi10Mg (**i**,**j**) and AlSi10Mg/10 vol.%Si_3_N_4_ (**k,l**)_,_ AlSi10Mg (**m**,**n**) and AlSi10Mg/1.5 wt.%TiC+1.5 wt.%TiB_2_ (**o**,**p**) (reproduced with permission from [79,93,95,104]).

**Figure 25:**
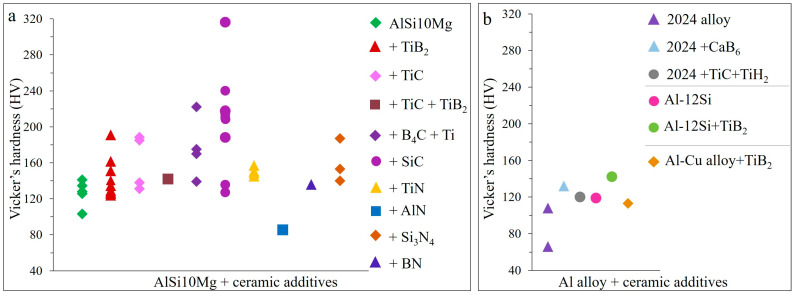
Hardness results of LPBF prepared ceramic particulate reinforced AlSi10Mg (**a**) and other Al alloys (**b**) (data from [11,13,28,31,64,70,71,73,77,78,80,82,83,89,90,92,93,94,95,96,98,99,100,101,102,103,104,105]).

**Figure 26:**
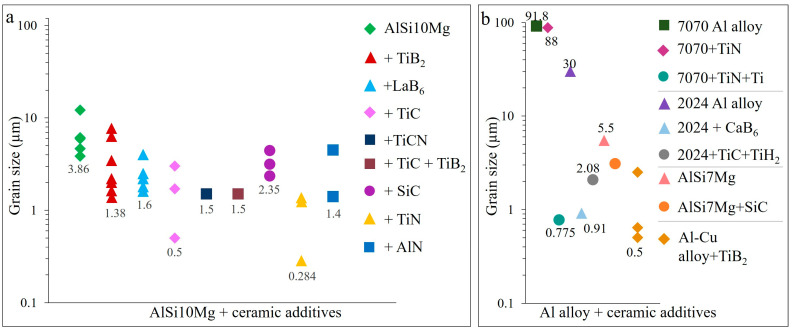
Average grain size of LPBF prepared ceramic particulate reinforced AlSi10Mg (**a**) and other Al alloys (**b**) (data from [8,28,31,50,59,66,67,73,77,78,79,80,81,82,83,84,88,91,92,93,96,99,100,101]).

The addition of TiB_2_ to the AlSi10Mg alloy results in fully dense samples with significantly refined grains (down to 0.5 µm), randomized crystallographic orientation, increased hardness up to 191 HV, tensile strength up to 540 MPa and elongation to 17.7% (Figure 23, Figure 24, Figure 25 and Figure 26). Similarly, high tensile strength is observed for the TiC/Al-15Si, double-reinforced TiC-TiB_2_/AlSi10Mg and hybrid TiN-Ti/7050 AMCs, however, with lower elongation (Figure 23a,b).

The tensile fracture of the AlSi10Mg-6.5 wt.%TiB_2_ composite showed that the fracture path of the AMC is not flat, as in the case of AlSi10Mg, but rather random for both horizontal and vertical samples (Figure 24a,b) [79]. Generally, the reinforced composites with refined microstructure have high ductility due to less stress concentration. Based on the fine-sized equiaxed dimples (Figure 24e,f), the failure mode of the AMC is a ductile fracture, stating improved ductility. However, the holes and the tears on the fracture surface might have led to premature failure of the AMC (Figure 24c,d). Similarly, in the AlSi10Mg-0.2 wt.%LaB_6_ composite, cracking predominantly occurred within the melt pool boundaries, and the LaB_6_ nanoparticles led to more ductile fracture of the composite, owing to fine equiaxed dimples [84]. Ductile-type failure was reported for AlSi10Mg with homogeneously dispersed circular-structured TiC (3 wt.%). The latter contributed to the improvement of tensile strength without sacrificing ductility [71]. The dual TiB_2_ and TiC reinforced AMC’s tensile fracture (Figure 24m,n) possesses fewer pores and deeper dimples as compared to AlSi10Mg (Figure 24o,p) and shows mixed ductile and brittle fracture mode. The relatively hard intragranular TiB_2_ and TiC particles accommodate the dislocations in the grains, contributing to strain hardening and uniform elongation [93]. Both brittle and ductile fractures were observed in the case of 0.7 wt.% hybrid Ti-B_4_C addition. However, the further increase in additive content led to fracture changes from ductile to brittle [94].

When analyzing SiC reinforced AlSi10Mg, huge attention was given to applied energy, as under low energy, brittle Al_4_C_3_ is formed. However, higher energy promotes the formation of Al_4_SiC_4_, along with a well-dispersed eutectic structure, hence prohibiting the premature failure of the composite [95]. Similar to SiC (Figure 24g,h), in Si_3_N_4_ reinforced AMC (Figure 24k,l), the nature of the fracture is ductile brittle, dominated by brittle, whereas pure AlSi10Mg (Figure 24i,j) shows a ductile-brittle composite fracture dominated by ductile. Due to Si_3_N_4_, crack propagation is suppressed when the tip meets the Si_3_N_4_–AlSi0Mg interface. However, because of the irregular distribution of Si_3_N_4_ and the changes in propagation path of the connected cracks, more cleavage steps were formed [104]. When TiN nanoparticles are added to AlSi10Mg, the fracture behavior of the alloy remains in mixed failure mode; however, large-size agglomerates formed during excess addition of TiN, decreasing both strength and ductility [101]. 

Analyses show that the highest hardness was shown by 15 wt.%SiC reinforced AMCs, followed by the 17.2 wt.% hybrid B_4_C-Ti and 11.6 wt.%TiB_2_ reinforced materials (Figure 25a). Hardness values of TiC and Si_3_N_4_ reinforced AMCs are comparable with TiB_2_. Meanwhile, ceramic reinforced 2024, Al–12Si and Al–Cu alloys show inferior hardness compared to AlS10Mg with similar additives (Figure 25b).

The AMCs reinforced with TiB_2_, TiC, hybrid TiN-Ti and TiC-TiH_2_ additives are subjected to in situ formation of L1_2_-Al_3_Ti or D0_22_-Al_3_Ti (Table 4), which serve as active nucleation sites and promote grain refinement in the 0.5–2 µm range (Figure 26a,b). The substantial grain refinement, down to submicron level, is achieved by the incorporation of TiN and CaB_6_ into AMCs, resulting in both significantly enhanced hardness and tensile strength (Figure 26a,b).

The degree of improvement depends on additive content and composition of the Al alloy. Table 4 briefly summarizes the influence of the reported ceramic additives on the LPBF process and their content limitation.

## 4. Summary and Outlook

LPBF technologies are now commercially available and attract a huge deal of attention in research community. Although the number of aluminum alloys suitable for AM through LPBF is quite limited, the process keeps evolving, and, in the nearest future, a widespread application of AM of high-strength aluminum alloys is expected to occur in the aerospace market.

The cost of industrial metal printers remains the chief capital expenditure of AM parts to achieve economies-of-scale cost reduction. Although the industry has suffered due to COVID-19, the reverse has now begun. In light of current metal printers’ high prices, they are mostly used in high-value industries, such as aerospace, defense and medical. Other fields, such as energy, are starting to show interest in powder bed fusion technology, although developing economically viable applications requires sufficient time.

**Figure 27:**
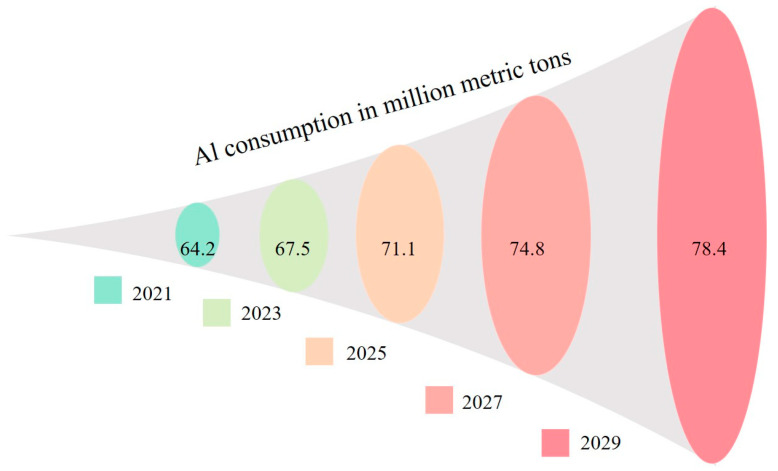
Calculated aluminum consumption up to 2029 (adapted from Ref. [113]).

A 2.6 percent annual growth rate is predicted for aluminum consumption globally up to 2029. In 2021, global aluminum consumption is projected at 64.2 million metric tons alone (Figure 27).

However, fuel efficiency and low carbon emission are the mantra for new-era airliners, which have groundbreaking design equipped with composite materials comprising 50 percent of the primary structure, hence eliminating the use of numerous aluminum parts [114]. In addition, the world’s biggest aluminum producers are limiting the production of Al, planning to reduce energy consumption and encourage the producers to develop green and low-carbon technologies and produce high-quality, high-strength and long-life aluminum products through innovations [115]. This means that there is a need for revolutionary actions to keep additive manufacturing of aluminum alloys on track.

Over the next decade, the development of new 3D printable Al alloys is expected to bring down the cost and enlarge the materials’ capacity and portfolio. For example, the lightweight aluminum–lithium alloys could contribute to reducing aircraft weight, also benefiting from excellent fatigue resistance and cryogenic toughness in addition to light weight and high specific modulus.

As numerous reinforcements are used to further enhance the properties of Al alloys, one big step ahead will be using different reinforcing particles (ceramics) and covering them with compatible coatings to provide suitable wettability and interface, or incorporating the reinforcing particles into Al alloy particles to provide a homogeneous distribution. Another main challenge is the recycling of the used feedstock and the utilization of the spattered debris to prepare new powders for further use.

As the design of new alloys applicable for the LPBF process is time and cost consuming, a high-throughput and reliable technique is needed to experimentally validate the custom alloys and effectively introduce them into the market. Therefore, a deep understanding of the impact of the alloying constituents on the processability of the feedstock by LPBF and, ultimately, the properties of the produced items in application, is of a crucial importance.

In this review paper, the effect of non-oxide ceramic (borides, nitrides, carbides) and hybrid reinforcing additives on the densification, grain refinement and respective mechanical characteristics of LPBF-fabricated AMCs was discussed. A comprehensive analysis of research studies on densification, compositional and microstructural characteristics of the in situ and ex situ reinforced aluminum alloys produced by LPBF method was accomplished to demonstrate the capability of different ceramic additives to tailor the mechanical properties with application to a wide variety of process parameters.

- Generally, an incorporation of the ceramic particles into Al alloys results in a significant improvement in strength, ductility and hardness of the fabricated parts accompanied by a refined microstructure and with randomization of crystallographic orientation of reinforced AMCs.
- Most of the AMCs can be densified to over 99% relative density; moreover, non-oxide ceramic additives significantly improve laser absorptivity of a powder feedstock.
- The addition of ceramic particulates shifts the process window to a higher energy regime; however, an applied excess energy may result in the evaporation or decomposition of ceramics particles (mainly SiC).
- The application of a laser re-melting strategy can further increase the densification degree and the surface quality of AMCs; however, it also can cause the evaporation and loss of ceramic particles.
- Hybrid reinforcements are proven to be the effective additives, providing the formation of a wide variety of reinforcing phases with a coherent interface with matrices.
- The use of ceramics with a fine-particle size results in an increased degree of densification, microstructural and compositional uniformity, as well as an apparent grain refinement.
- The addition of TiB_2_, CaB_6_, TiC, TiN to Al alloys leads to a considerable grain refinement, down to the submicron level, due to the intensive heterogeneous nucleation and grain growth inhibition.
- An addition of matching ceramics prevents the hot tearing and gives the prospect to consolidate crack-susceptible Al alloys by a laser powder-bed fusion technique.
- The highest elongation of 17.7% is demonstrated by the AlSi10Mg/TiB_2_ composite; however, the highest strength of 613 MPa is recorded for the hybrid TiN-Ti reinforced AMCs.
- The highest hardness of 316 HV is estimated for SiC reinforced AMCs, which possess a relatively high strength and moderate ductility.

## Figures and Tables

**Table 2:** Characteristics of carbide reinforced AMCs fabricated by laser powder-bed fusion.

System	Used Device, Process Parameters	Relative Density (%)	Average Grain Size (μm)	σ_y_/σ_u_ (MPa)	ε/ε_c_ (%)	Hardness (HV)	N
Al-15Si	SLM125 P = 360 W ν = 600 mm/s d = 20 µm h = 60 µm	>98.5	-	σ_u_ = 398	ε = 2.6	154 HV1	[89]
Al-15Si/ 1 wt.% TiC				σ_u_ = 578	ε = 7.86	146 HV1	
Al-15Si/ 2.5 wt.% TiC				σ_u_ ≈ 450	ε ≈ 4	150 HV1	
Al-15Si/ 10 wt.% TiC				σ_u_ ≈ 313	ε = 2.24	177 HV1	
AlSi10Mg/ 3 wt.% TiC	SLM system P = 80, 100, 120 and 140 W ν = 200 mm/s d = 50 µm h = 50 µm E = 160 J/mm^3^	>98.5	-	σ_u_ = 452	ε = 9.8	157.4 HV0.1	[71]
	E = 200 J/mm^3^			-	-	≈173 HV0.1	
	E = 240 J/mm^3^			σ_u_ = 486	ε = 10.9	188.3 HV0.1	
	E = 280 J/mm^3^			-	-	180.6 HV0.1	
AlSi10Mg/ 5 wt.% TiC	SLM system P = 110 W ν = 100–350 mm/s d = 50 µm h = 50 µm E_l_ = 1100, 733, 440, 314 J/m	>98	-	-	-	181.2 HV0.2	[70]
AlSi10Mg/ 5 wt.% TiC	EOS M290 P = 320 W ν = 1100 mm/s d = 30 µm h = 130 µm	99.75	0.5–1	σ_u_ ≈ 456 σ_y_ ≈ 338	ε = 2.97	131 HV0.05	[31]
AlSi10Mg/ 5 wt.% TiC	SLM system P = 100 W ν = 150 mm/s d = 50 µm h = 50 µm	Full dense	-	σ_u_ = 482	ε = 10.8	185 HV0.1	[90]
AlSi10Mg/ 10 wt.% Al-Ti-C-B master alloy	3D Systems ProX DMP 320 P = 300 W ν = 1400 mm/s d = 30 µm h = 100 µm	-	~3	σ_u_ = 488 ± 6 σ_y_ = 287 ± 3	ε = 10.1 ± 2.2	-	[88]
2024 alloy	EOS M290 P = 200 W ν = 100 mm/s d = 40 µm h = 90 µm T = 180 °C	98.2	~30	σ_u_ = 240 ± 10	ε = 0.3 ± 0.2	108 HV0.2	[92]
2024/ 1 wt.% TiC		98.5	-	-	-		
2024/ 1 wt.% TiH_2_		95.7	-	-	-	-	
2024/ (1 wt.% TiC +1 wt.% TiH_2_)		97.1	~2	σ_u_ = 390 ± 15	ε = 12.0 ± 0.5	120 HV0.2	
AlSi10Mg	EOS M280 P = 270 W ν = 1600 mm/s d = 30 µm h = 110 µm	98.22	12.1	σ_u_ = 393.8 ± 14.5 σ_y_ = 224.2 ± 7.2	ε = 4.5 ± 0.9	127.8 ± 2.4 HV.1	[93]
ASi10Mg/ 1.5 wt.% TiC +1.5 wt.% TiB_2_		99.02	1.5	σ_u_ = 552.4 ± 12.1 σ_y_ = 325 ± 10.2	ε = 12 ± 0.6	142 ± 2.9 HV0.1	
ASi10Mg/ 3 wt.% TiB_2_		97.12	7.7	σ_u_ = 360.6 ± 8.5 σ_y_ = 200 ± 8.8	ε = 3.8 ± 0.2	134.4 ± 1.4 HV0.1	
ASi10Mg/ 3 wt.% TiC		98.23	1.7	σ_u_ = 453 ± 10 σ_y_ = 267.5 ± 7.8	ε = 4.8 ± 1.1	138.3 ± 1.7 HV0.1	
AlSi10Mg	SLM-125HL P = 150 W ν = 1200 mm/s d = 30 µm h = 105 µm T = 200 °C	At RT full dense	-	RT σ_u_ = 356 ± 10 σ_y_ = 220 ± 4	ε = 4.5 ± 0.5	-	[91]
				100 °C σ_u_ = 327 ± 2 σ_y_ = 230 ± 3	ε = 5 ± 1		
				150 °C σ_u_ = 282 ± 3 σ_y_ = 213 ± 3	ε = 11.5 ± 2.5		
				200 °C σ_u_ = 245 ± 8 σ_y_ = 194 ± 7	ε = 11 ± 1.2		
AlSi10Mg/ 2 vol.% TiCN		At RT full dense	RT <1.5	RT σ_u_ = 333 ± 2 σ_y_ = 227 ± 7	ε = 2.8 ± 0.	-	
			-	100 °C σ_u_ = 344 ± 2 σ_y_ = 245 ± 2	ε = 3.5 ± 0.2		
			-	150 °C σ_u_ = 308 ± 9 σ_y_ = 235 ± 4	ε = 4.2 ± 0.2		
			-	200 °C σ_u_ = 270 ± 1 σ_y_ = 209 ± 10	ε = 4.9 ± 0.4		
AlSi10Mg	SLM-120 P = 200 W ν = 1200 mm/s d = 30 µm h = 70 µm T = 200 °C	Almost full dense	-	σ_u_ = 366 σ_y_ = 193	ε = 6.8	~141 HV0.2	[94]
AlSi10Mg/ 0.7 wt.% (B_4_C+Ti)				σ_u_ = 417 σ_y_ = 234	ε = 5.2	~139 HV0.2	
AlSi10Mg/ 5.7 wt.% (B_4_C+Ti)				σ_u_ = 307 σ_y_ = 126	ε = 3.6	~170 HV0.2	
AlSi10Mg/ 11.5 wt.% (B_4_C+Ti)				σ_u_ = 218 σ_y_ = 117	ε = 3.4	~175 HV0.2	
AlSi10Mg/ 17.2 wt.% (B_4_C+Ti)				σ_u_ = 165 σ_y_ = 72	ε = 1.7	~222 HV0.2	
AlSi7Mg	EOSINT M280 P = 350 W ν = 1200 mm/s d = 40 µm h = 190 µm T = 80 °C	Porosity ≈0.59%	~4.55	σ_u_ = 388.3 ± 49.6	ε = 7.03 ± 1.25	≈1.85 GPa nano-hardness	[8]
AlSi7Mg/ 2 wt.% SiC		Porosity ≈0.25%	~3.14	σ_u_ = 502.94	ε = 10.64 ± 1.06	≈2.11 GPa nano-hardness	
AlSi10Mg/ 2 vol.% SiC (~2.4 wt.%)	SLM280HL P = 120 W ν = 250 mm/s d = 30 µm h = 60 µm T = 150 °C E_v_ = 267 J/mm^3^	~92.04	-	-	-	-	[95]
	P = 150 W E_v_ = 333 J/mm^3^	98.7	4.44	σ_u_ = 343 ± 59	ε = 3.3 ± 1.7	134.4 ± 3.2 HV0.1	
	P = 180 W E_v_ = 400 J/mm^3^	97.69	4.96	σ_u_ = 377 ± 28	ε = 2.9 ± 0.95	135.6 ± 3.5 HV0.1	
	P = 210 W E_v_ = 467 J/mm^3^	97.36	6.73	σ_u_ = 440 ± 17	ε ≈ 7.4	131.7 ± 2.6 HV0.1	
	P = 240 W E_v_ = 533 J/mm^3^	97.40	-	σ_u_ = 450 ± 30	ε = 4.9	129.7 ± 6.9 HV0.1	
Al–12Si/ 10 vol.% SiC (~11.8 wt.%)	ReaLizer SLM-100 P = 200 W ν = 375–1500 mm/s d = 50 µm h = 100 µm E_v_ ≈ 20–80 J/mm^3^	97.4 (by X-ray micro tomography (XMT))	-	-	-	-	[34]
AlSi10Mg/ 10 wt.% SiC	EOSINT M280 P = 240–320 W ν = 500–1800 mm/s d = 30 µm h = 80–160 µm	-	2.35	σ_u_ ≈ 450 σ_y_ ≈ 410	-	208.5 HV0.1	[96]
AlSi10Mg/ 15 wt.% SiC	Self-developed NRD-SLM-III P = 340–490 W ν = 600–2100 mm/s d = 40 µm h = 60–180 µm T = 200 °C	97.7	-	σ_u_ = 341.9	ε ≈ 3	217.4 HV0.2	[97]
AlSi10Mg/ 15 wt.% SiCp (300 mesh)	Self-developed NRD-SLM-III P = 500 W ν = 1200 mm/s d = 40 µm h = 120 µm T = 200 °C	≈97.8	-	σ_uc_ = 545.4	ε_c_ ≈ 4.7%	≈210 HV0.2	[98]
AlSi10Mg/ 15 wt.% SiCp (600 mesh)		≈98.5		σ_uc_ = 642.4	ε_c_ ≈ 6.1%	≈240 HV0.2	
AlSi10Mg/ 15 wt% SiCp (1200 mesh)		98.9		σ_uc_ = 764.1	ε_c_ ≈ 7.0%	316.1 HV0.2	
AlSi10Mg/ 20 wt.% SiC	Self-developed P = 80–110 W Ν = 100 mm/s d = 50 µm h = 50 µm E_l_ = 800–1100 J/m	~89.2–96.1	-	-	-	214 HV0.1	[11]
AlSi10Mg/ 20 wt.% SiC D50_SiC_ = 50 μm	SLM apparatus with Yb laser P = 100 W ν = 100 mm/s d = 30 µm h = 50 µm	86.4	-	-	-	~127 HV0.1	[13]
AlSi10Mg/ 20 wt.% SiC D50_SiC_ = 15 μm		93.7				188 HV0.1	
AlSi10Mg/ 20 wt.% SiC D50_SiC_ = 5 μm		~97.2				218.5 HV0.1	

**Table 3:** Characteristics of nitride reinforced AMCs fabricated by laser powder-bed fusion.

System	Used Device, Process Parameters	Relative Density (%)	Average Grain Size (μm)	σ_y/_σ_u_ (MPa)	ε/ε_c_ (%)	Hardness (HV)	N
AlSi10Mg/ 2 wt.% TiN (D50_TiN_ = 80 nm)	Dimetal-80 SLM system P = 100 W ν = 200–600 mm/s d = 30 µm h = 80 µm	97.6	0.284	-	-	145 ± 4.9 HV0.1	[99,100]
AlSi10Mg	SLM-280 HL P = 100 W ν = 1200 mm/s d = 30 µm h = 90 µm	Porosity =0.9%	3.86	σ_u_ = 359.4 ± 8.5 σ_y_ = 264 ± 10.5	ε = 3.9 ± 0.3	134.6 ± 4.4 HV0.1	[101]
AlSi10Mg/ 2 wt.% TiN		Porosity =0.2%	1.37	σ_u_ = 386.1 ± 12.6 σ_y_ = 295.9 ± 4.6	ε = 4.4 ± 0.27	148.5 ± 4.1 HV0.1	
AlSi10Mg/ 4 wt.% TiN		Porosity =0.01%	1.24	σ_u_ = 491.8 ± 5.5 σ_y_ = 315.4 ± 5.2	ε = 7.5 ± 0.29	156.9 ± 4.9 HV0.1	
AlSi10Mg/ 6 wt.% TiN		Porosity =3.7%	1.19	σ_u_ = 325.1 ± 14.2 σ_y_ = 261.6 ± 3.5	ε = 2.9 ± 0.32	150.4 ± 3.1 HV0.1	
7050 Al alloy	SLM-280 HL P = 210 W ν = 115 mm/s d = 30 µm h = 50 µm	98.5	91.8	σ_u_ = 75 ± 25	ε ≈ 0.6	-	[66]
7050/0.18 wt.% TiN		98.9	88	σ_u_ = 111 ± 3	ε = 1.1 ± 0.2		
7050/0.36 wt.% TiN		-	-	σ_u_ ≈140	ε ≈ 1		
7050/0.54 wt.% TiN		-	-	σ_u_ ≈ 60	ε ≈ 0.9		
7050/1.82 wt.% Ti		99.6	2.3	σ_u_ = 427 ± 12	ε = 3.9 ± 1.1		
7050/3.64 wt.% Ti		-	-	σ_u_ ≈ 480	ε ≈ 6.1		
7050/5.46 wt.% Ti		-	-	σ_u_ ≈ 350	ε ≈ 2.5		
7050/2 wt.% (TiN+Ti)		99.7	0.775	σ_u_ ≈ 550	ε ≈ 8.6		
7050/4 wt.% (TiN+Ti)		-	-	σ_u_ = 613±15	ε = 8.8 ± 0.8		
7050/6 wt.% (TiN+Ti)		-	-	σ_u_ ≈ 408	ε ≈ 13.2		
AlSi10Mg/ 1 wt.% AlN (50 nm)	SLM apparatus P = 200 W ν = 100–300 mm/s d = 30 µm h = 60–100 µm E_v_ = 1100 J/mm^3^	97	4.5	-	-	-	[67]
	E_v_ = 660 J/mm^3^	60	2				
	E_v_ = 420 J/mm^3^	Full dense	1.4				
	E_v_ = 220 J/mm^3^	Full dense	2				
AlSi10Mg/ 2 wt.% AlN	Self-made P = 200 W ν = 100 mm/s d = 30 µm h = 80 µm	-	-	-	-	77–85.3 HV0.05	[102]
AlSi10Mg	EOSINT M290 P = 380 W ν = 1300 mm/s d = 30 µm h = 200 µm	Porosity =0.15%	-	σ_u_ ≈ 180	ε ≈ 5.6	103 HV0.2	[103]
AlSi10Mg/ 1 wt.% BN		Porosity =0.81%		σ_u_ = 230	ε ≈ 2.3	136 HV0.2	
AlSi10Mg	EOSINT M290 P = 180–300 W ν = 300–800 mm/s d = 30 µm h = 30–70 µm T = 150 °C	-	-	σ_u_ = 432 ± 15 σ_y_ = 275 ± 13	ε = 5.12 ± 0.29	128 ± 3 HV0.2	[104]
AlSi10Mg/ 5 vol.% Si_3_N_4_ (~5.8 wt.%)		99.49 ± 0.17		σ_u_ = 447 ± 18 σ_y_ = 308 ± 12	ε = 3.58 ± 0.15	140 ± 7 HV0.2	
AlSi10Mg/ 10 vol.% Si_3_N_4_ (~11.5 wt.%)		99.18 ± 0.16		σ_u_ = 485 ± 12 σ_y_ = 362 ± 18	ε = 2.47 ± 0.23	153 ± 3 HV0.2	
AlSi10Mg/ 15 vol.% Si_3_N_4_ (~17.1 wt.%)		98.41 ± 0.22		σ_u_ = 399 ± 21	ε = 0.66 ± 0.31	187 ± 13 HV0.2	

**Table 4:** The effect of reinforcing compounds on the fabrication and properties of AMCs and their optimal content limit.

Reinforcing Compound	Influence on the LPBF Process and the Properties of the Al Alloys	Minimum Optimal Limit
TiB_2_	Exhibits good wettability, interfacial compatibility with Al. Increases densification level, serves as grain refiner along with in situ formed Al_3_Ti, stabilizes grain boundaries, leads to randomized crystallographic orientation, dramatically improves strength, hardness and ductility.	2–6.5 wt.%
LaB_6_	Forms highly coherent interface with Al, leads to significant grain refinement, microstructural homogeneity, isotropic mechanical properties, does not have huge effect on strength enhancement, but improves ductility.	Up to 0.5 wt.%
CaB_6_	Serves as excellent grain refiner, microstructure stabilizer at the grain boundaries, forms highly coherent interface with Al, improves hardness, tensile strength, without sacrificing ductility.	Up to 2 wt.%
TiC	Using fine TiC particles leads to fully dense part fabrication with improved strength, ductility and hardness. The in situ formed D0_22_-Al_3_Ti inoculants provide heterogeneous nucleation of α-Al, leading to grain refinement, and remove the preferred orientation of the α-Al (200) phase. Depending on the TiC content and process parameters, novel circular (ring) structures are formed within the matrix, enhancing the mechanical performance of AMCs.	Up to 5 wt.%
TiC_B_	The gas-atomized powders release enormous TiC_B_ particles during LPBF process, largely promoting the nucleation of Al grains, grain refinement and resulting in weak crystallographic texture of AMCs. TiC_B_ particles along with precipitated Si enhance the yield strength, tensile strength and elongation.	~0.5 wt.%
TiCN	The addition of TiCN significantly reduces the average grain size, improves yield strength and ductility over native LPBF AlSi10Mg and rarely induces the formation of brittle Al_4_C_3_.	2 wt.%
TiC+TiH_2_	Due to decomposition of TiH_2_ and reaction of Al with Ti, a well-bonded interface between L1_2_-Al_3_Ti and α-Al was observed acting as substrate for α-Al heterogeneous nucleation. Meanwhile, the presence of Ti creates “Ti transition zone” between TiC and matrix, creating potent nucleation sites for α-Al as well. Owing to restriction of columnar grain growth, the joint effect of refinement strengthening, the reinforced AMCs exhibit enhanced mechanical performance, tensile strength and ductility.	1 wt.%TiC 1 wt.%TiH_2_
TiC+TiB_2_	Dual TiB_2_+TiC particles induce heterogeneous nucleation of Al and significantly refine the grains of the Al matrix. Double reinforcement results in simultaneous enhancement in strength, ductility and hardness, acting more efficiently than single species.	1.5 wt.%TiC 1.5 wt.%TiH_2_
SiC	Use of fine (nanosized or few-micron-sized) SiC results in grain refinement, decrease in porosity, enhancement of hardness, tensile strength and ductility but, depending on the process parameters, can cause in situ formation of Al_4_C_3_ or Al_4_SiC_4_ phases.	Up to 2 wt.%
Ti+B_4_C	In situ formed TiC, TiB_2_ and Ti_3_SiC_2_ serve as nucleants and reinforcements. The Ti+B_4_C content increase results in improvement in hardness, however much lower elongation and tensile strength. The released heat during the combustion reaction allows for fabricating the materials at low applied laser energy.	0.7 wt.%
Al_4_C_3_	Al_4_C_3_ itself is a brittle and unstable phase and is best avoided. However, small amounts of formed nanosized Al_4_C_3_ can enhance the mechanical properties of AMCs.	-
Al_4_SiC_4_	Al_4_SiC_4_ along with intermetallic Mg_2_Si increase reinforcement/matrix wettability and the resultant interfacial bonding coherence. Al_4_SiC_4_ serves as the transition zone, which hinders the direct contact of SiC and aluminum crystals. Ultrafine Al_4_SiC_4_ has a reinforcing effect, improving the mechanical properties of SiC reinforced AMCs.	-
TiN	TiN particles refine the α-Al grains due to intensive heterogeneous nucleation and increase the fraction of low-energy high-angle grain boundaries, enhancing the hardness and strength. Due to the Al+TiN reaction, Al_3.21_Si_0.47_ and a (Ti,Al)N graded layer is formed, which significantly enhances the hardness due to improving interface bonding strength. The coherent interfaces between the matrix, Mg_2_Si and TiN particles lead to precipitation strengthening, which contributes to the overall strength increase.	4 wt.%
TiN+Ti	Provides crack-free microstructure and significant grain refinement due to formation of Al_3_Ti phase and different precipitates, improves the hardness and tensile strength.	4 wt.%
AlN	The AlN particles show high chemical stability and good compatibility with Al alloy. They promote densification, refine the α-Al grains, create strain-hardened tribo-layer, enhancing the wear resistance and stabilizing the coefficient of friction.	1 wt.%
BN	The formation of AlN and AlB_2_ phases during the solid-state reaction of Al+BN results in increased tensile strength and hardness, though at the expense of porosity increase. However, increase in BN content and particle size decreases wettability and prevents uniform metal spreading.	1 wt.%
Si_3_N_4_	Si_3_N_4_ particles increase the melt pool’s viscosity and disturb the stability, suggesting a much narrower window for LPBF process parameters. Owing to hindered dislocation motion during deformation (because of difference of Al and Si_3_N_4_) and the load-bearing effect of Si_3_N_4_ particles, the AMCs possess improved strength and elastic modulus.	10 vol.%

## Data Availability

The data supporting the findings of this study is available within the article.

## References

[1] Spierings A.B., Dawson K., Uggowitzer P.J., Wegener K. (2018). Influence of SLM scan-speed on microstructure, precipitation of Al3Sc particles and mechanical properties in Sc- and Zr-modified Al-Mg alloys. Mater. Des..

[2] Otani Y., Sasaki S. (2020). Effects of the addition of silicon to 7075 aluminum alloy on microstructure, mechanical properties, and selective laser melting processability. Mater. Sci. Eng. A.

[3] Muhammad M., Nezhadfar P., Thompson S., Saharan A., Phan N., Shamsaei N. (2021). A comparative investigation on the microstructure and mechanical properties of additively manufactured aluminum alloys. Int. J. Fatigue.

[4] Li P., Li R., Yang H., Yuan T., Niu P., Wang M., Li L., Chen C. (2020). Selective laser melting of Al-3.48Cu-2.03Si-0.48Sc-0.28Zr alloy: Microstructure evolution, properties and metallurgical defects. Intermetallics.

[5] Qian W., Zhao Y., Kai X., Yan Y., Gao X., Jin L. (2020). Microstructure and properties of 6111Al matrix composites reinforced by the cooperation of in situ ZrB2 particles and Y. J. Alloys Compd..

[6] Qbau N., Nam N., Hien N., Ca N. (2020). Development of light weight high strength aluminum alloy for selective laser melting. J. Mater. Res. Technol..

[7] Totten G.E., Tiryakioğlu M., Kessler O. (2018). Encyclopedia of Aluminum and Its Alloys.

[8] Wang M., Song B., Wei Q., Shi Y. (2019). Improved mechanical properties of AlSi7Mg/nano-SiCp composites fabricated by selective laser melting. J. Alloys Compd..

[9] Tan Q., Fan Z., Tang X., Yin Y., Li G., Huang D., Zhang J., Liu Y., Wang F., Wu T. (2021). A novel strategy to additively manufacture 7075 aluminium alloy with selective laser melting. Mater. Sci. Eng. A.

[10] Zhang J., Song B., Wei Q., Bourell D., Shi Y. (2018). A review of selective laser melting of aluminum alloys: Processing, microstructure, property and developing trends. J. Mater. Sci. Technol..

[11] Gu D., Chang F., Dai D. (2015). Selective Laser Melting Additive Manufacturing of Novel Aluminum Based Composites With Multiple Reinforcing Phases. J. Manuf. Sci. Eng..

[12] Famodimu O.H., Stanford M., Oduoza C.F., Zhang L. (2018). Effect of process parameters on the density and porosity of laser melted AlSi10Mg/SiC metal matrix composite. Front. Mech. Eng..

[13] Chang F., Gu D., Dai D., Yuan P. (2015). Selective laser melting of in-situ Al_4_SiC_4_ + SiC hybrid reinforced Al matrix composites: Influence of starting SiC particle size. Surf. Coatings Technol..

[14] Zhou L., Huynh T., Park S., Hyer H., Mehta A., Song S., Bai Y., McWilliams B., Cho K., Sohn Y. (2020). Laser powder bed fusion of Al–10 wt% Ce alloys: Microstructure and tensile property. J. Mater. Sci..

[15] Wallis C., Buchmayr B., Bermejo R., Supancic P. (2020). Fabrication of 3D metal-ceramic (Al-AlN) architectures using laser-powder bed fusion process. Addit. Manuf..

[16] Minasyan T., Aghayan M., Liu L., Aydinyan S., Kollo L., Hussainova I., Rodríguez M.A. (2018). Combustion synthesis of MoSi2 based composite and selective laser sintering thereof. J. Eur. Ceram. Soc..

[17] Minasyan T., Ivanov R., Toyserkani E., Hussainova I. (2021). Laser powder-bed fusion of Mo(Si,Al)_2_—Based composite for elevated temperature applications. J. Alloys Compd..

[18] Wang J., Liu T., Luo L., Cai X., Wang B., Zhao J., Cheng Z., Wang L., Su Y., Xue X. (2021). Selective laser melting of high-strength TiB2/AlMgScZr composites: Microstructure, tensile deformation behavior, and mechanical properties. J. Mater. Res. Technol..

[19] Minasyan T., Ivanov R., Toyserkani E., Hussainova I. (2021). Mo(Si,Al)_2_ by laser powder bed fusion of AlSi10Mg and combustion synthesized MoSi2. Mater. Lett..

[20] Minasyan T., Aydinyan S., Toyserkani E., Hussainova I. (2020). Parametric Study on In Situ Laser Powder Bed Fusion of Mo(Si_1−x_,Al_x_)_2_. Materials.

[21] Kuai Z., Li Z., Liu B., Liu W., Yang S. (2022). Effects of remelting on the surface morphology, microstructure and mechanical properties of AlSi10Mg alloy fabricated by selective laser melting. Mater. Chem. Phys..

[22] Gu D., Yuan P. (2015). Thermal evolution behavior and fluid dynamics during laser additive manufacturing of Al-based nanocomposites: Underlying role of reinforcement weight fraction. J. Appl. Phys..

[23] Yu W., Sing S., Chua C., Kuo C., Tian X. (2019). Particle-reinforced metal matrix nanocomposites fabricated by selective laser melting: A state of the art review. Prog. Mater. Sci..

[24] Kumar M.B., Sathiya P. (2020). Methods and materials for additive manufacturing: A critical review on advancements and challenges. Thin-Walled Struct..

[25] Bayat M., Nadimpalli V.K., Pedersen D.B., Hattel J.H. (2020). A fundamental investigation of thermo-capillarity in laser powder bed fusion of metals and alloys. Int. J. Heat Mass Transf..

[26] Martin J.H., Yahata B.D., Hundley J.M., Mayer J.A., Schaedler T.A., Pollock T.M. (2017). 3D printing of high-strength aluminium alloys. Nature.

[27] Griffiths S., Rossell M.D., Croteau J., Vo N.Q., Dunand D.C., Leinenbach C. (2018). Effect of laser rescanning on the grain microstructure of a selective laser melted Al-Mg-Zr alloy. Mater. Charact..

[28] Mair P., Goettgens V.S., Rainer T., Weinberger N., Letofsky-Papst I., Mitsche S., Leichtfried G. (2021). Laser powder bed fusion of nano-CaB6 decorated 2024 aluminum alloy. J. Alloys Compd..

[29] Zhou L., Hyer H., Chang J., Mehta A., Huynh T., Yang Y., Sohn Y. (2021). Microstructure, mechanical performance, and corrosion behavior of additively manufactured aluminum alloy 5083 with 0.7 and 1.0 wt% Zr addition. Mater. Sci. Eng. A.

[30] Plotkowski A., Sisco K., Bahl S., Shyam A., Yang Y., Allard L., Nandwana P., Rossy A.M., Dehoff R. (2020). Microstructure and properties of a high temperature Al–Ce–Mn alloy produced by additive manufacturing. Acta Mater..

[31] Fan Z., Yan X., Fu Z., Niu B., Chen J., Hu Y., Chang C., Yi J. (2021). In situ formation of D022-Al3Ti during selective laser melting of nano-TiC/AlSi10Mg alloy prepared by electrostatic self-assembly. Vacuum.

[32] Kaufman J.G. Introduction to Aluminum Alloys and Tempers. https://books.google.ee/books?hl=en&lr=&id=idmZIDcwCykC&oi=fnd&pg=PR7&dq=Introduction+to+Aluminum+Alloys+and+Tempers&ots=YF2Do8uYO4&sig=lVfaG-D2QRHLKNTb8Nivh0VpmmA&redir_esc=y#v=onepage&q=Introduction%20to%20Aluminum%20Alloys%20and%20Tempers&f=false.

[33] Montero-Sistiaga M.L., Mertens R., Vrancken B., Wang X., Van Hooreweder B., Kruth J.-P., Van Humbeeck J. (2016). Changing the alloy composition of Al7075 for better processability by selective laser melting. J. Mater. Process. Technol..

[34] Astfalck L., Kelly G.K., Li X., Sercombe T.B. (2017). On the Breakdown of SiC during the Selective Laser Melting of Aluminum Matrix Composites. Adv. Eng. Mater..

[35] Li X., Wang X., Saunders M., Suvorova A., Zhang L., Liu Y., Fang M., Huang Z., Sercombe T. (2015). A selective laser melting and solution heat treatment refined Al–12Si alloy with a controllable ultrafine eutectic microstructure and 25% tensile ductility. Acta Mater..

[36] Zhuo L., Wang Z., Zhang H., Yin E., Wang Y., Xu T., Li C. (2018). Effect of post-process heat treatment on microstructure and properties of selective laser melted AlSi10Mg alloy. Mater. Lett..

[37] Wang M., Song B., Wei Q., Zhang Y., Shi Y. (2018). Effects of annealing on the microstructure and mechanical properties of selective laser melted AlSi7Mg alloy. Mater. Sci. Eng. A.

[38] Zhang C., Zhu H., Hu Z., Zhang L., Zeng X. (2019). A comparative study on single-laser and multi-laser selective laser melting AlSi10Mg: Defects, microstructure and mechanical properties. Mater. Sci. Eng. A.

[39] Li W., Li S., Liu J., Zhang A., Zhou Y., Wei Q., Yan C., Shi Y. (2016). Effect of heat treatment on AlSi10Mg alloy fabricated by selective laser melting: Microstructure evolution, mechanical properties and fracture mechanism. Mater. Sci. Eng. A.

[40] Ji Y., Dong C., Kong D., Li X. (2020). Design materials based on simulation results of silicon induced segregation at AlSi10Mg interface fabricated by selective laser melting. J. Mater. Sci. Technol..

[41] Wang Y., Shi J. (2020). Effect of hot isostatic pressing on nanoparticles reinforced AlSi10Mg produced by selective laser melting. Mater. Sci. Eng. A.

[42] Bi J., Lei Z., Chen Y., Chen X., Tian Z., Lu N., Qin X., Liang J. (2020). Microstructure, tensile properties and thermal stability of AlMgSiScZr alloy printed by laser powder bed fusion. J. Mater. Sci. Technol..

[43] Thapliyal S., Shukla S., Zhou L., Hyer H., Agrawal P., Agrawal P., Komarasamy M., Sohn Y., Mishra R.S. (2021). Design of heterogeneous structured Al alloys with wide processing window for laser-powder bed fusion additive manufacturing. Addit. Manuf..

[44] Lu J., Lin X., Kang N., Cao Y., Wang Q., Huang W. (2021). Keyhole mode induced simultaneous improvement in strength and ductility of Sc modified Al–Mn alloy manufactured by selective laser melting. Mater. Sci. Eng. A.

[45] Thapliyal S., Komarasamy M., Shukla S., Zhou L., Hyer H., Park S., Sohn Y., Mishra R.S. (2019). An integrated computational materials engineering-anchored closed-loop method for design of aluminum alloys for additive manufacturing. Materialia.

[46] Yang K., Shi Y., Palm F., Wu X., Rometsch P. (2018). Columnar to equiaxed transition in Al-Mg(-Sc)-Zr alloys produced by selective laser melting. Scr. Mater..

[47] Kurnsteiner P., Bajaj P., Gupta A., Benjamin W., Weisheit A., Li X., Leinebach C., Gault B., Jagle E., Raabe D. (2020). Control of thermally stable core-shell nano-precipitates in additively manufactured Al-Sc-Zr alloys. Addit. Manuf..

[48] Zhou L., Hyer H., Thapliyal S., Mishra R.S., McWilliams B., Cho K., Sohn Y. (2020). Process-Dependent Composition, Microstructure, and Printability of Al-Zn-Mg and Al-Zn-Mg-Sc-Zr Alloys Manufactured by Laser Powder Bed Fusion. Met. Mater. Trans. A.

[49] Zhou S., Su Y., Wang H., Enz J., Ebel T., Yan M. (2020). Selective laser melting additive manufacturing of 7xxx series Al-Zn-Mg-Cu alloy: Cracking elimination by co-incorporation of Si and TiB_2_. Addit. Manuf..

[50] Biffi C.A., Bassani P., Fiocchi J., Albu M., Tuissi A. (2021). Selective laser melting of AlCu-TiB2 alloy using pulsed wave laser emission mode: Processability, microstructure and mechanical properties. Mater. Des..

[51] Jia Q., Rometsch P., Kürnsteiner P., Chao Q., Huang A., Weyland M., Bourgeois L., Wu X. (2019). Selective laser melting of a high strength Al Mn Sc alloy: Alloy design and strengthening mechanisms. Acta Mater..

[52] Kang N., El Mansori M., Lin X., Guittonneau F., Liao H., Huang W., Coddet C. (2018). In-situ synthesis of aluminum/nano-quasicrystalline Al-Fe-Cr composite by using selective laser melting. Compos. Part B Eng..

[53] Kang N., Fu Y., Coddet P., Guelorget B., Liao H., Coddet C. (2017). On the microstructure, hardness and wear behavior of Al-Fe-Cr quasicrystal reinforced Al matrix composite prepared by selective laser melting. Mater. Des..

[54] Demir A.G., Previtali B. (2017). Multi-material selective laser melting of Fe/Al-12Si components. Manuf. Lett..

[55] Aboulkhair N.T., Simonelli M., Parry L., Ashcroft I., Tuck C., Hague R. (2019). 3D printing of Aluminium alloys: Additive Manufacturing of Aluminium alloys using selective laser melting. Prog. Mater. Sci..

[56] Mair P., Braun J., Kaserer L., March L., Schimbäck D., Letofsky-Papst I., Leichtfried G. (2022). Unique microstructure evolution of a novel Ti-modified Al-Cu alloy processed using laser powder bed fusion. Mater. Today Commun..

[57] Wang Z., Wang X., Chen X., Qiu C. (2022). Complete columnar-to-equiaxed transition and significant grain refinement in an aluminium alloy by adding Nb particles through laser powder bed fusion. Addit. Manuf..

[58] Tan Q., Zhang J., Mo N., Fan Z., Yin Y., Bermingham M., Liu Y., Huang H., Zhang M.-X. (2020). A novel method to 3D-print fine-grained AlSi10Mg alloy with isotropic properties via inoculation with LaB6 nanoparticles. Addit. Manuf..

[59] Xiao Y., Bian Z., Wu Y., Ji G., Li Y., Li M., Lian Q., Chen Z., Addad A., Wang H. (2019). Effect of nano-TiB2 particles on the anisotropy in an AlSi10Mg alloy processed by selective laser melting. J. Alloys Compd..

[60] Kotadia H., Gibbons G., Das A., Howes P. (2021). A review of Laser Powder Bed Fusion Additive Manufacturing of aluminium alloys: Microstructure and properties. Addit. Manuf..

[61] Wang L., Jue J., Xia M., Guo L., Yan B., Gu D. (2016). Effect of the Thermodynamic Behavior of Selective Laser Melting on the Formation of In situ Oxide Dispersion-Strengthened Aluminum-Based Composites. Metals.

[62] Minasyan T., Aydinyan S., Liu L., Volubujeva O., Toyserkani E., Hussainova I. (2020). Mo(Si_1−x_,Al_x_)_2_-based composite by reactive laser powder-bed fusion. Mater. Lett..

[63] Dadbakhsh S., Mertens R., Hao L., Van Humbeeck J., Kruth J. (2018). Selective Laser Melting to Manufacture “In Situ” Metal Matrix Composites: A Review. Adv. Eng. Mater..

[64] Xi L., Wang P., Prashanth K., Li H., Prykhodko H., Scudino S., Kaban I. (2019). Effect of TiB2 particles on microstructure and crystallographic texture of Al-12Si fabricated by selective laser melting. J. Alloys Compd..

[65] Macías J.G.S., Douillard T., Zhao L., Maire E., Pyka G., Simar A. (2020). Influence on microstructure, strength and ductility of build platform temperature during laser powder bed fusion of AlSi10Mg. Acta Mater..

[66] Li X., Li G., Zhang M.-X., Zhu Q. (2021). Novel approach to additively manufacture high-strength Al alloys by laser powder bed fusion through addition of hybrid grain refiners. Addit. Manuf..

[67] Dai D., Gu D., Xia M., Ma C., Chen H., Zhao T., Hong C., Gasser A., Poprawe R. (2018). Melt spreading behavior, microstructure evolution and wear resistance of selective laser melting additive manufactured AlN/AlSi10Mg nanocomposite. Surf. Coatings Technol..

[68] Wang P., Eckert J., Prashanth K.-G., Wu M.-W., Kaban I., Xi L.-X., Scudino S. (2020). A review of particulate-reinforced aluminum matrix composites fabricated by selective laser melting. Trans. Nonferrous Met. Soc. China.

[69] Tjong S.C. (2007). Novel Nanoparticle-Reinforced Metal Matrix Composites with Enhanced Mechanical Properties. Adv. Eng. Mater..

[70] Gu D., Wang H., Chang F., Dai D., Yuan P., Hagedorn Y.-C., Meiners W. (2014). Selective Laser Melting Additive Manufacturing of TiC/AlSi10Mg Bulk-form Nanocomposites with Tailored Microstructures and Properties. Phys. Procedia.

[71] Gu D., Wang H., Dai D., Yuan P., Meiners W., Poprawe R. (2015). Rapid fabrication of Al-based bulk-form nanocomposites with novel reinforcement and enhanced performance by selective laser melting. Scr. Mater..

[72] Gu D., Wang H., Dai D., Chang F., Meiners W., Hagedorn Y.-C., Wissenbach K., Kelbassa I., Poprawe R. (2015). Densification behavior, microstructure evolution, and wear property of TiC nanoparticle reinforced AlSi10Mg bulk-form nanocomposites prepared by selective laser melting. J. Laser Appl..

[73] Xi L., Gu D., Guo S., Wang R., Ding K., Prashanth K.G. (2020). Grain refinement in laser manufactured Al-based composites with TiB2 ceramic. J. Mater. Res. Technol..

[74] Kusoglu I.M., Gökce B., Barcikowski S. (2020). Use of (nano-)additives in Laser Powder Bed Fusion of Al powder feedstocks: Research directions within the last decade. Procedia CIRP.

[75] Liu L., Minasyan T., Ivanov R., Aydinyan S., Hussainova I. (2020). Selective laser melting of TiB2-Ti composite with high content of ceramic phase. Ceram. Int..

[76] Gu D., Yang Y., Xi L., Yang J., Xia M. (2019). Laser absorption behavior of randomly packed powder-bed during selective laser melting of SiC and TiB_2_ reinforced Al matrix composites. Opt. Laser Technol..

[77] Xiao Y., Chen H., Bian Z., Sun T., Ding H., Yang Q., Wu Y., Lian Q., Chen Z., Wang H. (2021). Enhancing strength and ductility of AlSi10Mg fabricated by selective laser melting by TiB_2_ nanoparticles. J. Mater. Sci. Technol..

[78] Xi L., Guo S., Gu D., Guo M., Lin K. (2019). Microstructure development, tribological property and underlying mechanism of laser additive manufactured submicro-TiB_2_ reinforced Al-based composites. J. Alloys Compd..

[79] Feng Z., Tan H., Fang Y., Lin X., Huang W. (2021). Selective laser melting of TiB_2_/AlSi10Mg composite: Processability, microstructure and fracture behavior. J. Mater. Process. Technol..

[80] Li X., Ji G., Chen Z., Addad A., Wu Y., Wang H., Vleugels J., Van Humbeeck J., Kruth J. (2017). Selective laser melting of nano-TiB_2_ decorated AlSi10Mg alloy with high fracture strength and ductility. Acta Mater..

[81] Wang P., Gammer C., Brenne F., Niendorf T., Eckert J., Scudino S. (2018). A heat treatable TiB_2_/Al-3.5Cu-1.5Mg-1Si composite fabricated by selective laser melting: Microstructure, heat treatment and mechanical properties. Compos. Part B Eng..

[82] Mair P., Kaserer L., Braun J., Weinberger N., Letofsky-Papst I., Leichtfried G. (2020). Microstructure and mechanical properties of a TiB2-modified Al–Cu alloy processed by laser powder-bed fusion. Mater. Sci. Eng. A.

[83] Xi L.X., Zhang H., Wang P., Li H.C., Prashanth K.G., Lin K.J., Kaban I., Gu D.D. (2018). Comparative investigation of microstructure, mechanical properties and strengthening mechanisms of Al-12Si/TiB2 fabricated by selective laser melting and hot pressing. Ceram. Int..

[84] Tan Q., Yin Y., Fan Z., Zhang J., Liu Y., Zhang M.-X. (2020). Uncovering the roles of LaB6-nanoparticle inoculant in the AlSi10Mg alloy fabricated via selective laser melting. Mater. Sci. Eng. A.

[85] Wearing D., Horsfield A.P., Xu W., Lee P.D. (2016). Which wets TiB_2_ inoculant particles: Al or Al_3_Ti?. J. Alloys Compd..

[86] Savalani M., Ng C., Li Q., Man H. (2012). In situ formation of titanium carbide using titanium and carbon-nanotube powders by laser cladding. Appl. Surf. Sci..

[87] Masanta M., Shariff S., Choudhury A.R. (2011). Evaluation of modulus of elasticity, nano-hardness and fracture toughness of TiB_2_–TiC–Al_2_O_3_ composite coating developed by SHS and laser cladding. Mater. Sci. Eng. A.

[88] Gao T., Zhang S., Liu G., Sun Q., Liu J., Sun Q., Sun J., Wang Z., Liu X., Wang X. (2021). A high strength AlSi10Mg alloy fabricated by laser powder bed fusion with addition of Al Ti C B master alloy powders. Materialia.

[89] Zhou Y., Wen S., Wang C., Duan L., Wei Q., Shi Y. (2019). Effect of TiC content on the Al-15Si alloy processed by selective laser melting: Microstructure and mechanical properties. Opt. Laser Technol..

[90] Wang H., Gu D. (2014). Nanometric TiC reinforced AlSi10Mg nanocomposites: Powder preparation by high-energy ball milling and consolidation by selective laser melting. J. Compos. Mater..

[91] He P., Kong H., Liu Q., Ferry M., Kruzic J.J., Li X. (2021). Elevated temperature mechanical properties of TiCN reinforced AlSi10Mg fabricated by laser powder bed fusion additive manufacturing. Mater. Sci. Eng. A.

[92] Liu X., Liu Y., Zhou Z., Wang K., Zhan Q., Xiao X. (2021). Grain refinement and crack inhibition of selective laser melted AA2024 aluminum alloy via inoculation with TiC–TiH_2_. Mater. Sci. Eng. A.

[93] Cheng W., Liu Y., Xiao X., Huang B., Zhou Z., Liu X. (2021). Microstructure and mechanical properties of a novel (TiB_2_+TiC)/AlSi_10_Mg composite prepared by selective laser melting. Mater. Sci. Eng. A.

[94] Yi J., Zhang X., Rao J.H., Xiao J., Jiang Y. (2020). In-situ chemical reaction mechanism and non-equilibrium microstructural evolution of (TiB_2_ + TiC)/AlSi10Mg composites prepared by SLM-CS processing. J. Alloys Compd..

[95] Wang Z., Zhuo L., Yin E., Zhao Z. (2021). Microstructure evolution and properties of nanoparticulate SiC modified AlSi10Mg alloys. Mater. Sci. Eng. A.

[96] Zhang D., Yi D., Wu X., Liu Z., Wang W., Poprawe R., Schleifenbaumc J.H., Zieglerd S. (2021). SiC reinforced AlSi10Mg composites fabricated by selective laser melting. J. Alloys Compd..

[97] Xue G., Ke L., Zhu H., Liao H., Zhu J., Zeng X. (2019). Influence of processing parameters on selective laser melted SiCp/AlSi10Mg composites: Densification, microstructure and mechanical properties. Mater. Sci. Eng. A.

[98] Xue G., Ke L., Liao H., Chen C., Zhu H. (2020). Effect of SiC particle size on densification behavior and mechanical properties of SiCp/AlSi10Mg composites fabricated by laser powder bed fusion. J. Alloys Compd..

[99] Gao C., Xiao Z., Liu Z., Zhu Q., Zhang W. (2018). Selective laser melting of nano-TiN modified AlSi10Mg composite powder with low laser reflectivity. Mater. Lett..

[100] Gao C., Wang Z., Xiao Z., You D., Wong K., Akbarzadeh A. (2020). Selective laser melting of TiN nanoparticle-reinforced AlSi10Mg composite: Microstructural, interfacial, and mechanical properties. J. Mater. Process. Technol..

[101] Gao C., Wu W., Shi J., Xiao Z., Akbarzadeh A. (2020). Simultaneous enhancement of strength, ductility, and hardness of TiN/AlSi10Mg nanocomposites via selective laser melting. Addit. Manuf..

[102] Dai D., Gu D., Poprawe R., Xia M. (2017). Influence of additive multilayer feature on thermodynamics, stress and microstructure development during laser 3D printing of aluminum-based material. Sci. Bull..

[103] Konopatsky A.S., Kvashnin D.G., Corthay S., Boyarintsev I., Firestein K.L., Orekhov A., Arkharova N., Golberg D.V., Shtansky D.V. (2021). Microstructure evolution during AlSi10Mg molten alloy/BN microflake interactions in metal matrix composites obtained through 3D printing. J. Alloys Compd..

[104] Miao K., Zhou H., Gao Y., Deng X., Lu Z., Li D. (2021). Laser powder-bed-fusion of Si3N4 reinforced AlSi10Mg composites: Processing, mechanical properties and strengthening mechanisms. Mater. Sci. Eng. A.

[105] Rauchenecker J., Rabitsch J., Schwentenwein M., Konegger T. (2021). Additive manufacturing of aluminum nitride ceramics with high thermal conductivity via digital light processing. Open Ceram..

[106] Li Q., Wang Z., Wu C., Cheng X. (2015). Microstructure and mechanical properties of aluminum nitride co-doped with cerium oxide via hot-pressing sintering. J. Alloys Compd..

[107] Dai D., Gu D. (2016). Influence of thermodynamics within molten pool on migration and distribution state of reinforcement during selective laser melting of AlN/AlSi10Mg composites. Int. J. Mach. Tools Manuf..

[108] Gu D., Ma C., Xia M., Dai D., Shi Q. (2017). A Multiscale Understanding of the Thermodynamic and Kinetic Mechanisms of Laser Additive Manufacturing. Engineering.

[109] Xue Y., Jiang X., Bourgeois L., Dai P., Mitome M., Zhang C., Yamaguchi M., Matveev A., Tang C., Bando Y. (2015). Aluminum matrix composites reinforced with multi-walled boron nitride nanotubes fabricated by a high-pressure torsion technique. Mater. Des..

[110] Minasyan T., Liu L., Aghayan M., Kollo L., Kamboj N., Aydinyan S., Hussainova I. (2018). A novel approach to fabricate Si_3_N_4_ by selective laser melting. Ceram. Int..

[111] Minasyan T., Liu L., Aghayan M., Rodriguez M.A., Aydinyan S., Hussainova I. (2019). Mesoporous fibrous silicon nitride by catalytic nitridation of silicon. Prog. Nat. Sci..

[112] Minasyan T., Liu L., Holovenko Y., Aydinyan S., Hussainova I. (2019). Additively manufactured mesostructured MoSi_2_-Si_3_N_4_ ceramic lattice. Ceram. Int..

[113] Worldwide Aluminum Consumption Forecast 2029|Statista. https://www.statista.com/statistics/863681/global-aluminum-consumption/.

[114] Boeing: First of Many: 787 Dreamliner Celebrates 10 Years since First Flight. https://www.boeing.com/features/2019/12/787-1st-flight-anniversary-12-19.page.

[115] China Sets Carbon Reduction Plans for Steel, Aluminium|Argus Media. https://www.argusmedia.com/en/news/2266222-china-sets-carbon-reduction-plans-for-steel-aluminium.

